# Loss of heat shock factor initiates intracellular lipid surveillance by actin destabilization

**DOI:** 10.1016/j.celrep.2022.111493

**Published:** 2022-10-18

**Authors:** Abigail Watterson, Sonja L.B. Arneaud, Naureen Wajahat, Jordan M. Wall, Lexus Tatge, Shaghayegh T. Beheshti, Melina Mihelakis, Nicholas Y. Cheatwood, Jacob McClendon, Atossa Ghorashi, Ishmael Dehghan, Chase D. Corley, Jeffrey G. McDonald, Peter M. Douglas

**Affiliations:** 1 Department of Molecular Biology, University of Texas Southwestern Medical Center, Dallas, TX 75390, USA; 2 Hamon Center for Regenerative Science and Medicine, UT Southwestern Medical Center, Dallas, TX 75390, USA; 3 Center for Human Nutrition, University of Texas Southwestern Medical Center, Dallas, TX 75390, USA; 4 Department of Molecular Genetics, University of Texas Southwestern Medical Center, Dallas, TX 75390, USA; 5 Lead contact

## Abstract

Cells sense stress and initiate response pathways to maintain lipid and protein homeostasis. However, the interplay between these adaptive mechanisms is unclear. Herein, we demonstrate how imbalances in cytosolic protein homeostasis affect intracellular lipid surveillance. Independent of its ancient thermo-protective properties, the heat shock factor, HSF-1, modulates lipid metabolism and age regulation through the metazoan-specific nuclear hormone receptor, NHR-49. Reduced *hsf-1* expression destabilizes the *Caenorhabditis elegans* enteric actin network, subsequently disrupting Rab GTPase-mediated trafficking and cell-surface residency of nutrient transporters. The ensuing malabsorption limits lipid availability, thereby activating the intracellular lipid surveillance response through vesicular release and nuclear translocation of NHR-49 to both increase nutrient absorption and restore lipid homeostasis. Overall, cooperation between these regulators of cytosolic protein homeostasis and lipid surveillance ensures metabolic health and age progression through actin integrity, endocytic recycling, and lipid sensing.

## In brief

Watterson et al. describe a mechanism through which HSF-1 affects lipid homeostasis. Through its ability to stabilize the enteric actin network, HSF-1 promotes membrane trafficking, cellular absorption, and lipid accumulation. Loss of *hsf-1* activates the intracellular lipid surveillance pathway via NHR-49, thereby linking cytosolic protein homeostasis with lipid metabolism.

## Graphical abstract

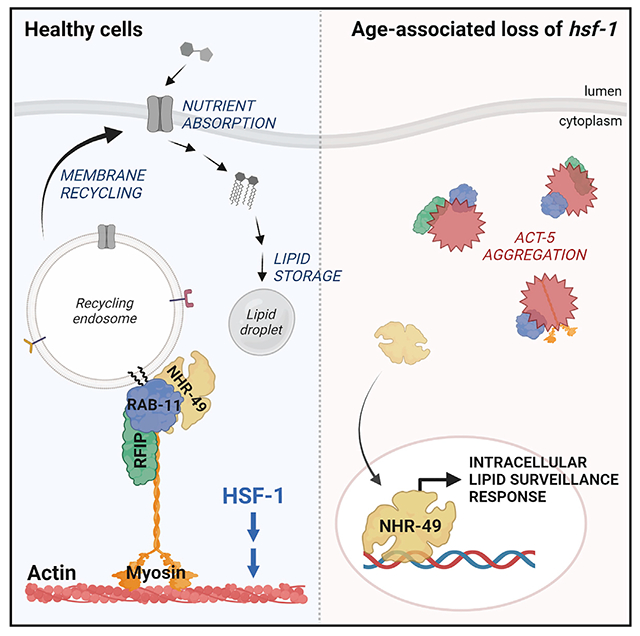

## INTRODUCTION

Comprising two of the four major biological macromolecules, proteins and lipids play critical roles across all kingdoms of life. To maintain cellular homeostasis in the face of an ever-changing environment, cells have evolved adaptive mechanisms to sense and respond to protein or lipid imbalances. In the context of lipid homeostasis, cells regulate membrane fluidity through homeoviscous adaptation and intracellular lipid deposition through tight coordination of anabolic and catabolic processes (Greenberg et al., 2011; Sinensky, 1974; Walther and Farese, 2012). Limiting sterol lipid availability such as cholesterol compromises both membrane dynamics and steroidogenesis (Dufourc, 2008; Miller and Bose, 2011). In response, cells promote lipogenesis through the sterol regulatory element binding protein (SREBP) to replenish the respective sterols and restore membrane fluidity (Brown and Goldstein, 1997; Levental et al., 2020). When cellular resources become limited, cells activate lipolysis and β-oxidation to mobilize and metabolize lipid reserves for energy production through nuclear receptors such as Hepatic Nuclear Factor 4 (HNF4) or Peroxisome Proliferator-Activated Receptor (PPAR) (Hayhurst et al., 2001; Reddy and Hashimoto, 2001). With respect to protein homeostasis, cells respond to proteome destabilization by activating organelle-specific signaling cascades to promote protein folding as well as modulate protein translation and degradation (Akerfelt et al., 2010; Shpilka and Haynes, 2018; Walter and Ron, 2011).

While adaptive mechanisms to restore protein or lipid homeostasis are seemingly distinct, their interplay has become increasingly apparent, particularly within the endoplasmic reticulum (ER). In response to imbalances in lipid bilayer composition or widespread protein misfolding within the ER lumen, cells activate the unfolded protein response (UPR) to alter both protein and lipid homeostasis (Covino et al., 2018; Promlek et al., 2011; Volmer et al., 2013). Along with EIF2α-mediated translational repression and enhanced chaperone-mediated protein folding, ER stress activates lipogenesis, alters lipid saturation, and expands organellular surface area for homeostatic restoration (Metcalf et al., 2020). Interplay between lipid and protein homeostasis at the ER appear bidirectional, as ER lipid bilayer stress caused by imbalances in sterol levels can block protein translocation into the ER (Nilsson et al., 2001). Conversely, XBP1-mediated UPR activation by protein misfolding has been reported to regulate hepatic lipogenesis genes (Lee et al., 2008). Although activation of the UPR is indicative of cell stress and has been linked with various disease states (Wang and Kaufman, 2012), its ectopic activation has been reported to prolong lifespan and mitigate progression of neurodegeneration (Scheper and Hoozemans, 2015; Taylor and Dillin, 2013). Coordination between lipid and protein homeostasis pathways plays an important role within the ER, yet their interplay in other subcellular locales is not well defined.

The cytosol offers potential for interactions between these different homeostatic mechanisms. While later stages of *de novo* lipid synthesis predominately occur in the ER (Jacquemyn et al., 2017), early steps of these biosynthetic pathways occur within the cytosol (Tracey et al., 2018). In parallel, most proteins in the cell are translated within the cytosol (Cross et al., 2009), with a subset being co-translationally transported into the ER. To accommodate compartmental differences, protein homeostasis throughout the cytosol is coordinated through distinct quality control mechanisms (Akerfelt et al., 2010). As the most ancestral means of protein homeostasis, cytosolic maintenance of proteome integrity amid thermal destabilization occurs through the transcriptional activation of protein folding enzymes and associated machinery by σ^32^ in bacteria or the heat shock transcription factor, HSF1, in protozoa (Abravaya et al., 1992; Grossman et al., 1984; Taylor et al., 1984). In metazoans, HSF1 serves a similar cytoprotective function but has adopted additional roles in development, metabolism, and age regulation that appear to be largely independent of its ancestral role in thermotolerance (Akerfelt et al., 2010; Baird et al., 2014; Brunquell et al., 2016; Douglas et al., 2015; Hsu et al., 2003; Morley and Morimoto, 2004). Constitutive activation of HSF-1 protects against thermal stress and extends lifespan in *Caenorhabditis elegans* (Baird et al., 2014). Conversely, age-associated reduction in HSF-1 activity disrupts the enteric actin network and compromises intestinal barrier integrity due to accelerated loss of epithelial cell junctions (Egge et al., 2019). In parallel, reducing *hsf1* expression in mammalian models decreases steady-state levels of metabolic enzymes involved in lipogenesis (Cigliano et al., 2017), and increasing reports highlight the involvement of HSF1 in modulating sterol and isoprenoid synthesis via the mevalonate pathway (Kang et al., 2019; Krishnamurthy et al., 2016; Su et al., 2019). Moreover, transcription of genes involved in fatty acid β-oxidation is activated upon reduced *hsf-1* expression in *C. elegans* (Brunquell et al., 2016). In a reciprocal fashion, reducing fatty acid synthesis inhibits basal HSF-1 activity (Kim et al., 2016). While emerging studies have linked this well-established regulator of cytosolic proteome integrity with lipid metabolism, the mechanistic relationship between these homeostatic pathways in the cytosol remains unclear.

As the transcriptional initiator of fatty acid β-oxidation in *C. elegans* (Van Gilst et al., 2005a, 2005b), the nuclear hormone receptor, NHR-49, is a critical regulator of intracellular lipid surveillance (Watterson et al., 2022). In the presence of ample intracellular lipids, NHR-49 is sequestered to endocytic vesicles within the cytosol via geranylgeranylation of the small G protein, RAB-11.1. Upon intracellular lipid depletion, the *de novo* synthesis and subsequent availability of a key lipid sensor, geranylgeranyl pyrophosphate, becomes limited, thereby preventing NHR-49 vesicular sequestration and driving its nuclear translocation. In the nucleus, NHR-49 activates transcription of β-oxidation genes as well as an inducible regulator of endocytic recycling, *rab-11.2*, to increase nutrient intake and restore lipid homeostasis (Watterson et al., 2022). While this lipid-sensing pathway has been linked with conditions of lipid depletion, its ability to affect or be affected by protein quality control pathways has not been described. Through modulating HSF-1 levels in *C. elegans*, we investigated how this regulator of protein folding influences lipid homeostasis in relation to cellular and organismal health. Herein, we describe how mechanisms of protein homeostasis and lipid surveillance are integrated within the cytosol.

## RESULTS

### HSF-1 affects lipid deposition and metabolism in *C. elegans*

**Figure 1.**
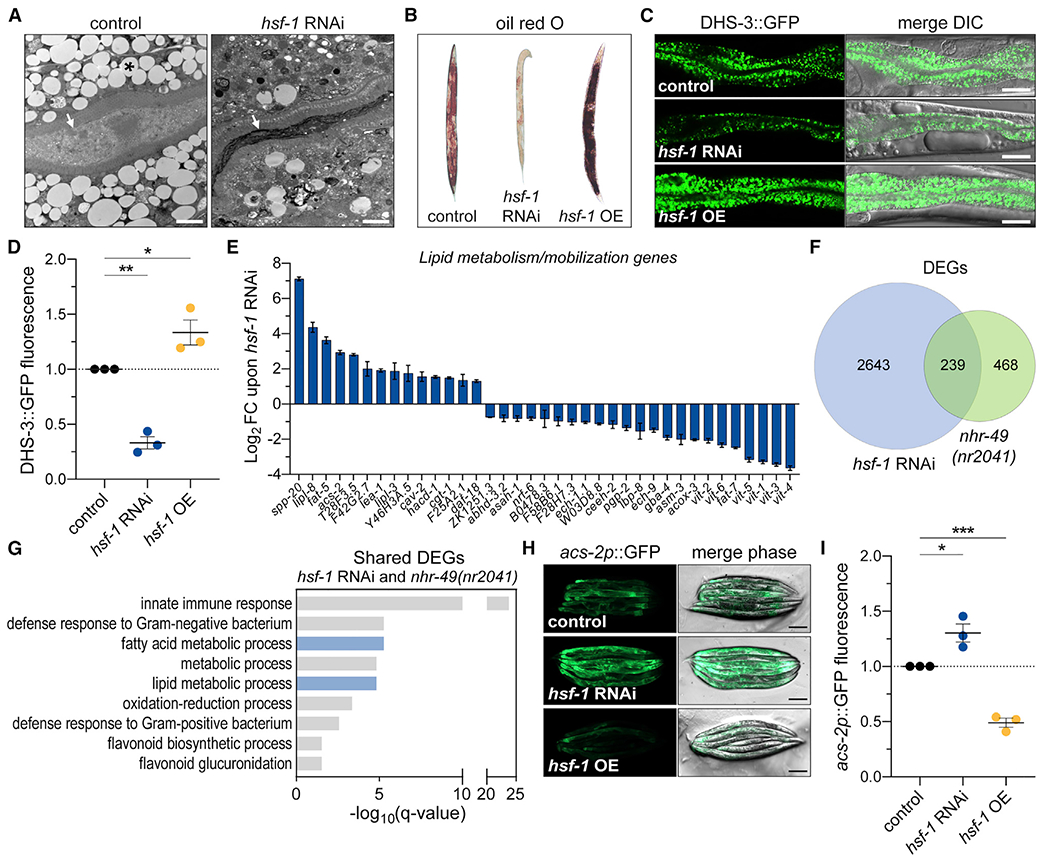
HSF-1 affects lipid deposition and modulates metabolism gene transcripts through NHR-49 (A) Transmission electron microscopy (TEM) images of intestinal cross sections from adult *C. elegans* treated with empty vector control or *hsf-1* RNAi. Micrographs show lumen and adjacent enterocytes; arrows mark lumen, asterisk marks lipid droplet. Scale bar, 5 μm. (B–D) Lipid deposition in day 5 adult wild-type control, *hsf-1* RNAi-treated, and *hsf-1*-overexpression (*hsf-1* OE) *C. elegans*. (B) Representative micrographs of oil red O staining. Representative micrographs (C) and relative fluorescence by flow cytometry (D) of intestinal lipid droplet marker, DHS-3::GFP. Scale bar, 25 μm. Data are mean ± SEM from three replicate experiments. *p = 0.0307, **p = 0.0011 by one-way ANOVA with Dunnett’s multiple comparisons. (E) Significantly regulated genes involved in lipid/fatty acid metabolism and mobilization upon *hsf-1* RNAi. Values represent log_2_ fold change (FC) transcript abundance, detected by RNA sequencing (RNA-seq), relative to control RNAi. See Table S1 for statistical significance. (F) Overlap of differentially expressed genes (DEGs) upon *hsf-1* RNAi, left, or the *nhr-49(nr2041)* mutation, right, compared with controls. See also Table S2. (G) Gene ontology (GO) term analysis of the shared DEGs upon *hsf-1* RNAi and the *nhr-49(nr2041)* mutation. Plot displays the most over-represented biological processes (based on false discovery rate [FDR]). Metabolic processes of interest highlighted in blue. (H and I) Fluorescence of the *acs-2p*::GFP transcriptional reporter in control, *hsf-1* RNAi, or *hsf-1* OE conditions. (H) Fluorescence micrographs. Scale bar, 200 mm. (I) Relative fluorescence by flow cytometry. Mean ± SEM for three replicate experiments. *p = 0.0118, ***p = 0.0009 by one-way ANOVA with Dunnett’s multiple comparisons. See also Figure S1 and Tables S1 and S2.

The intestine is the major metabolic tissue in *C. elegans* and serves as its primary lipid repository, similar to adipose tissue (Ashrafi et al., 2003). Ultrastructural analysis revealed a dramatic reduction of intestinal lipid droplets in animals treated with *hsf-1* RNAi (Figures 1A, S1A, and S1B). We confirmed that *hsf-1* RNAi reduced neutral triglyceride levels through staining with the lysochrome diazo dye, oil red O (Figures 1B and S1C). Conversely, overexpression of *hsf-1* in all tissues increased lipid accumulation in adult animals (Figures 1B and S1C). Moreover, fluorescence intensity of the medium-chain dehydrogenase lipid droplet marker, DHS-3::GFP, directly correlated with HSF-1 levels (Figures 1C and 1D). While this phenotype has not been reported in unicellular organisms, HSF-1 affects lipid accumulation in *C. elegans*, and emerging studies have shown that modulating HSF1 levels can affect lipid metabolism in mammalian systems (Cigliano et al., 2017; Kang et al., 2019; Krishnamurthy et al., 2016; Su et al., 2019). In the budding yeast, *Saccharomyces cerevisiae*, reduced *hsf1* expression (Breslow et al., 2008) did not affect lipid droplet abundance, as determined by monodansylpentane staining (Figures S1D and S1E). Thus, HSF-1 modulates intracellular lipid levels in *C. elegans*, while preliminary evidence in yeast suggests that this adapted role for HSF-1 may be specific for multicellular organisms.

To understand how HSF-1 affects fat accumulation, we examined its ability to affect transcription of lipid metabolism genes. Utilizing previously reported datasets (Baird et al., 2014; Brunquell et al., 2016), we confirmed numerous genes involved in β-oxidation and lipid mobilization are significantly regulated by *hsf-1* RNAi (Figure 1E; Table S1). However, a majority (68%) of these transcripts were not significantly changed in worms overexpressing *hsf-1*, and only 19% (7/37) were inversely regulated compared with *hsf-1* RNAi (Figure S1F; Table S1). Moreover, chromatin immunoprecipitation sequencing (ChIP-seq) analysis showed no HSF-1 binding to the 5′ promoter elements of select genes differentially expressed upon *hsf-1* RNAi, including the acyl-CoA synthetase, *acs-2*, and the stearoyl-CoA desaturases, *fat-5* and *fat-7*, when compared with heat shock-inducible molecular chaperones, *hsp-70, hsp-16.2*, and *hsp-16.41* (Li et al., 2016) (Figure S1G). These data suggest that the transcriptional changes in lipid metabolism genes observed upon *hsf-1* RNAi are likely due to fluctuations in lipid deposition or availability rather than direct transcriptional regulation by HSF-1.

### HSF-1 modulates β-oxidation through the metazoan-specific NHR-49

To identify the molecule(s) responsible for these transcriptional changes, we examined known regulators of lipid catabolism. Many of the transcripts differentially regulated by *hsf-1* RNAi are mutually regulated by NHR-49, which initiates β-oxidation in response to starvation (Van Gilst et al., 2005a, 2005b). Genome-wide transcriptomics revealed a significant overlap among the transcripts differentially expressed in *nhr-49(nr2041)* loss-of-function mutants and animals treated with *hsf-1* RNAi (Figure 1F; Table S2). In addition to innate immunity (Naim et al., 2021), metabolic processes involving fatty acids and lipids were enriched by Gene Ontology (GO) term analysis of the shared differentially expressed genes in these datasets (Figure 1G). While *hsf-1* RNAi modulated expression of several NHR-49 transcriptional targets, the *nhr-49(nr2041)* mutation did not affect expression of ancestral heat shock targets (Figure S1H). Additionally, activation of the *hsp-16.2p*::*GFP* transcriptional reporter upon heat shock was unchanged by *nhr-49* RNAi (Figures S1I and S1J). These data suggest that HSF-1 functions independently of NHR-49, while loss of *hsf-1* appears to promote NHR-49 activity. In support, *hsf-1* RNAi elevated fluorescence of the established NHR-49 transcriptional reporter, *acs-2p*::GFP (Burkewitz et al., 2015), in an NHR-49-dependent manner (Figures 1H, 1I, S1K, and S1L). Conversely, *acs-2p*::GFP fluorescence was reduced in worms overexpressing *hsf-1* (Figures 1H and 1I). Therefore, levels of HSF-1 affect the transcriptional activity of NHR-49.

### HSF-1 requires NHR-49 for lipid metabolism and age determination but not thermal adaptation

**Figure 2.**
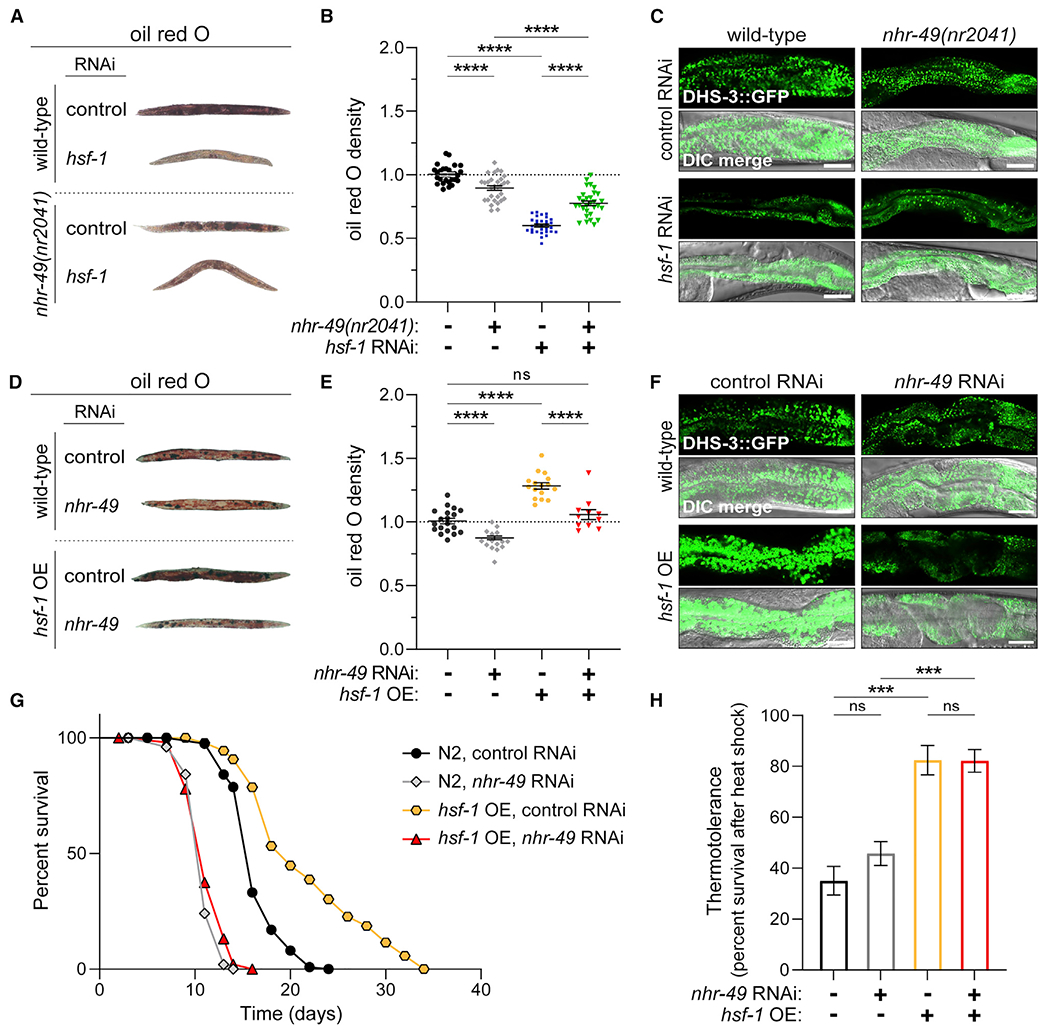
HSF-1 requires NHR-49 for lipid deposition and age determination but not thermal adaptation (A–C) Lipid deposition in day 5 adult wild-type and *nhr-49(nr2041)* mutant *C. elegans* treated with control or *hsf-1* RNAi. Representative micrographs (A) and relative density (B) of oil red O staining. Mean ± SEM for 30 animals per condition across three replicate experiments. ****p < 0.0001 by two-way ANOVA with Tukey’s multiple comparisons. (C) Representative micrographs of DHS-3::GFP expression. Scale bar, 25 μm. (D and E) Lipid deposition in day 5 adult wild-type and *hsf-1* OE *C. elegans* treated with control or *hsf-1* RNAi. Representative micrographs (D) and relative density (E) of oil red O staining. Mean ± SEM for, from left to right, 19, 15, 16, and 11 animals across three replicate experiments. ****p < 0.0001 by two-way ANOVA with Tukey’s multiple comparisons. (F) Representative micrographs of DHS-3::GFP expression. Scale bar, 25 μm. (G) Lifespan curves for N2 (wild type) and *hsf-1* OE worms cultured on control or *nhr-49* RNAi. Shown are representative lifespans of three replicates with approximately 100 animals per condition (see Table S3). p < 0.0001 by log rank test. (H) Percentage survival of N2 or *hsf-1* OE *C. elegans* treated with control or *nhr-49* RNAi and subjected to heat shock at 34°C for 12 h. Mean ± SEM for three replicate experiments. ns, not significant; ***p = 0.0009 by two-way ANOVA with Tukey’s multiple comparisons. See also Figure S2 and Table S3.

Unlike HSF1 and its primitive ancestor, σ^32^, nuclear hormone receptors first emerged in metazoans, as clear homologs are not present in protists, algae, or fungi (Escriva et al., 1998; Sladek, 2011). Considering their distinct origins, we were intrigued by the potential interplay between these transcriptional regulators. We sought to determine whether HSF-1 may have adapted to the emergence of this new class of nuclear receptor molecules and expanded its physiologic role in multicellular organisms. To understand the relationship between HSF-1 and NHR-49, we investigated whether NHR-49 affects the changes in lipid deposition observed upon modulation of *hsf-1* levels. While lipid deposition is elevated in *nhr-49(nr2041)* mutants during L4 larval stage (Van Gilst et al., 2005a), NHR-49 is required for age-associated maintenance of fat stores in adult animals (Ratnappan et al., 2014). In day 5 adult animals, lipid depletion by *hsf-1* RNAi was lessened in *nhr-49(nr2041)* mutants, as shown by oil red O staining and fluorescence intensity of DHS-3::GFP (Figures 2A–2C and S2A). In animals overexpressing *hsf-1*, we utilized *nhr-49* RNAi due to synthetic sterility in worms overexpressing *hsf-1* in the *nhr-49(nr2041)* mutant background. Consistent with NHR-49 being required for age-associated maintenance of fat stores (Ratnappan et al., 2014), increased lipid accumulation by *hsf-1* overexpression was abrogated upon *nhr-49* RNAi (Figures 2D–2F and S2B). Therefore, modulation of lipid deposition by HSF-1 requires NHR-49.

We next sought to determine whether this evolved role for HSF-1 in lipid metabolism also corresponds with its ability to modulate the aging process. While elevated HSF1 activity in *S. cerevisiae* does not alter replicative lifespan (Harris et al., 2001), *hsf-1* overexpression in *C. elegans* prolongs animal lifespan (Hsu et al., 2003; Morley and Morimoto, 2004). Despite lifespan extension by metabolic perturbations such as dietary restriction, reduced insulin signaling, and reduced mitochondrial respiration (Kenyon, 2010), impaired lipid metabolism in *nhr-49(nr2041)* mutants shortens lifespan (Van Gilst et al., 2005a). Shortened lifespan by *nhr-49* RNAi was comparable with *hsf-1* RNAi, and co-administering both RNAi was not additive, suggesting HSF-1 and NHR-49 act through a similar pathway (Figure S2C; Table S3). Conversely, lifespan extension by *hsf-1* overexpression (Baird et al., 2014) was abolished upon *nhr-49* RNAi (Figure 2G; Table S3), suggesting HSF-1 requires NHR-49 for the metabolic adaptation needed for lifespan extension. Thus, the ability of HSF-1 to initiate a metabolic response through NHR-49 plays an important role in both lipid deposition and age determination.

In unicellular organisms, HSF1 is critical for thermotolerance and maintenance of protein homeostasis (Morimoto et al., 1992). In *C. elegans*, HSF-1 functionality has expanded to include lipid metabolism and age determination through its interplay with NHR-49. Since the evolutionary emergence of NHR-49 occurred after HSF-1, we investigated whether thermal adaptation by HSF-1 functions independently of NHR-49. In accordance, young adult worms on *nhr-49* RNAi showed comparable levels of survival to prolonged heat stress when compared with control conditions (Figure 2H). Moreover, enhanced thermal protection by *hsf-1* overexpression was not affected by *nhr-49* RNAi (Figure 2H). Thus, the ancient role of HSF-1 as a mediator of thermal adaptation remains independent of NHR-49, yet emergent roles of HSF-1 in lipid metabolism and age regulation require this evolutionarily distinct nuclear receptor.

### Loss of HSF-1 activates the intracellular lipid surveillance response

**Figure 3.**
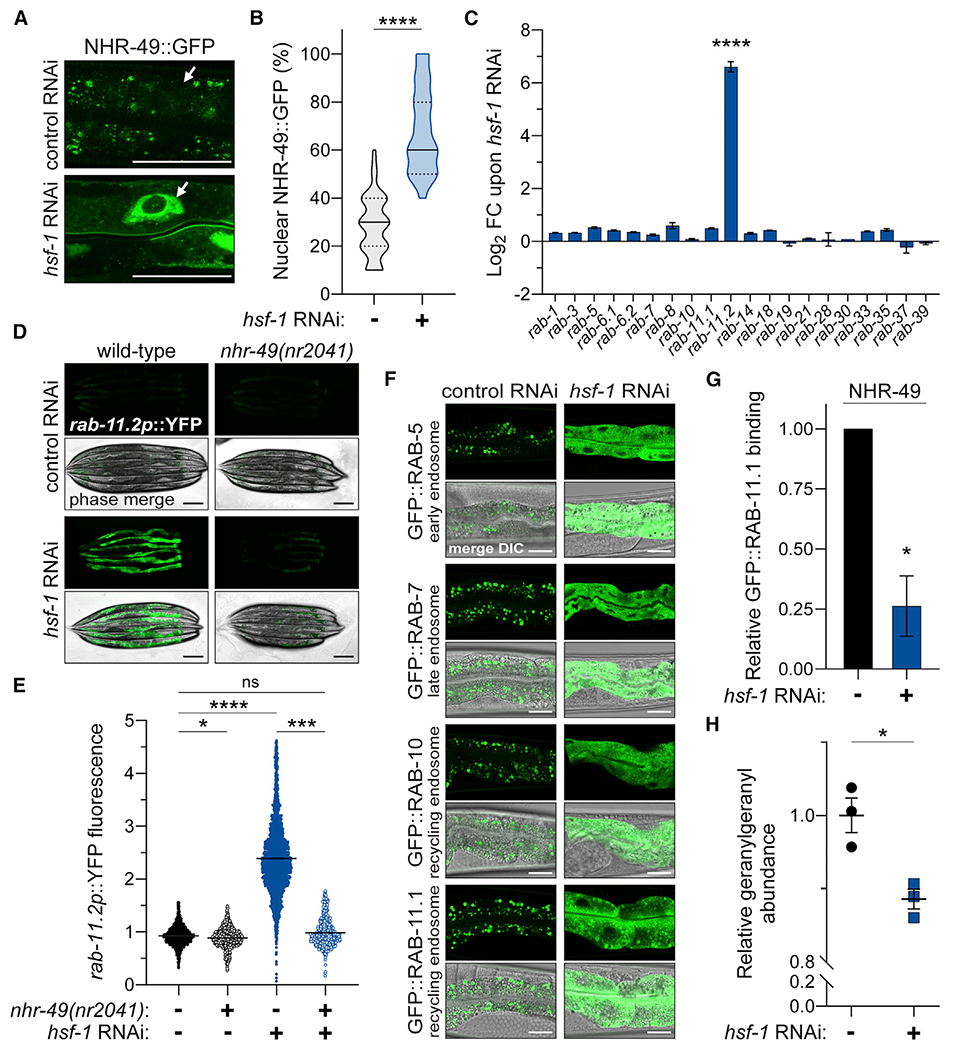
Loss of *hsf-1* activates the NHR-49-dependent intracellular lipid surveillance response (A–H) Comparative analyses of adult *C. elegans* treated with control or *hsf-1* RNAi.(A and B) NHR-49:GFP localization in the intestines of day 1 adults. (A) Representative micrographs; white arrows mark nuclear membrane. Scale bar, 10 μm. (B) Percentage of intestinal epithelia with nuclear-enriched NHR-49::GFP. Median with quartiles for 50 animals per condition from three replicate experiments. ****p < 0.0001 by two-tailed unpaired t test. (C) FC (log_2_) of Rab GTPase transcript abundance (based on reads per kilobase million [RPKM], detected by RNA-seq) upon *hsf-1* RNAi relative to control. Shown are mean ± SEM for two replicate experiments. ****p < 0.0001 by one-way ANOVA with Dunnett’s multiple comparisons. (D and E) Fluorescence of the *rab-11.2p*::YFP transcriptional reporter in adult wild-type and *nhr-49(nr2041)* mutant animals. (D) Fluorescence micrographs. Scale bar, 200 μm. (E) Relative fluorescence by flow cytometry. Mean ± SEM for (from left to right) 2,759; 3,355; 1,258; and 912 animals across three replicate experiments. ns, not significant; *p = 0.0157, ***p = 0.003, ****p < 0.0001 by two-way ANOVA with Tukey’s multiple comparisons. (F) Representative micrographs of GFP::RAB GTPase localization in the intestinal epithelia. Transgenic proteins, GFP:RAB-5, GFP::RAB-7, GFP::RAB-10, and GFP::RAB-11.1, decorate early, late, or recycling endocytic vesicles, respectively. Scale bar, 25 μm. (G) Relative NHR-49 enrichment in GFP::RAB-11.1 co-immunoprecipitation, detected by LC-MS/MS. Mean ± SEM for two biological replicates. *p = 0.0278 by two-tailed unpaired t test. (H) Relative geranylgeranyl levels measured from adult *C. elegans* cultured at 25°C. Mean ± SEM for three biological replicates. *p = 0.0135 by two-tailed unpaired t test. See also Figure S3.

We next sought to elucidate the molecular events underlying the interplay between these two transcriptional regulators. Due to the activation of NHR-49 transcriptional targets upon *hsf-1* RNAi, we hypothesized that loss of *hsf-1* initiates lipid catabolism through NHR-49 to accommodate energetic demand caused by lipid depletion. Thus, we investigated whether loss of *hsf-1* activates the intracellular lipid surveillance response in which lipid depletion promotes NHR-49 nuclear translocation and transcriptional activity (Watterson et al., 2022). NHR-49 serves as an important surveyor of intracellular lipid availability through its cytosolic sequestration to endocytic transport vesicles via the geranylgeranylation of endocytic recycling regulator, RAB-11.1. In conditions of lipid depletion, reduced *de novo* synthesis of the geranylgeranyl pyrophosphate lipid impairs Rab GTPase vesicular binding and promotes NHR-49 nuclear translocation. In the nucleus, NHR-49 activates expression of genes involved in β-oxidation as well as the small G protein, *rab-11.2*, to restore lipid homeostasis through increased nutrient absorption and metabolism. In accordance with transcriptomic data (Figure 1), *hsf-1* RNAi promoted the nuclear accumulation of NHR-49:GFP (Figures 3A and 3B). Moreover, *rab-11.2* was upregulated approximately 100-fold upon *hsf-1* RNAi, while transcription of other Rab GTPases was unchanged (Figure 3C). We confirmed by qPCR that *rab-11.2* but not *rab-11.1* transcripts were elevated upon *hsf-1* RNAi (Figure S3A). Moreover, the *rab-11.2* transcriptional reporter, *rab-11.2p*::YFP, confirmed its NHR-49-dependent activation in the intestinal epithelium by *hsf-1* RNAi (Figures 3D and 3E). Thus, loss of *hsf-1* promotes the nuclear accumulation and transcriptional activity of NHR-49.

The ability for HSF-1 to affect the nucleocytoplasmic distribution and activity of NHR-49 prompted us to examine how loss of *hsf-1* affects Rab GTPase dynamics since the geranylgeranylation status of these small G proteins plays a critical role in the cytosolic sequestration and inactivation of NHR-49 (Watterson et al., 2022). Utilizing GFP-tagged Rab GTPases within the intestine to monitor early (RAB-5), late (RAB-7), and recycling (RAB-10 and RAB-11.1) endosomes, we observed that *hsf-1* RNAi disrupted the association of these GTPases with transport vesicles, as shown by their diffuse cytosolic distribution (Figures 3F and S3B). Analysis of GFP::RAB-11.1 interactions in *hsf-1* RNAi versus control conditions through immunoprecipitation and peptide identification by liquid chromatography-tandem mass spectrometry (LC-MS/MS) demonstrated its reduced association with other annotated endocytic proteins as well as NHR-49 upon *hsf-1* RNAi (Figures 3G and S3C). Consistent with our previous findings that reduced geranylgeranyl synthesis and conjugation to RAB-11.1 prevents NHR-49 association with endocytic vesicles (Watterson et al., 2022), targeted lipidomics detected a significant loss in geranylgeranyl levels as early as day 1 of adulthood in worms treated with *hsf-1* RNAi (Figure 3H). Altogether, these data suggest that loss of *hsf-1* activates NHR-49 through the intracellular lipid surveillance pathway.

### Adaptive response to impaired apical recycling and malabsorption

By coupling β-oxidation with cellular absorption, the intracellular lipid surveillance response enables starving cells to both replenish and metabolize extrinsic resources, in part through *rab-11.2* activation. The Rab11 subfamily of small GTPases controls endocytic recycling of cell-surface proteins, including receptors, channels, and transporters (Welz et al., 2014). In conditions of metabolic demand, cells could increase their intake of extracellular resources by elevating the residency and recycling of nutrient transporters at the apical intestine. In addition to *rab-11.2* upregulation, *hsf-1* RNAi induced transcriptional upregulation of several apical cell-surface proteins, including the caveolin lipid transporter, *cav-2*; the P-glycoprotein efflux transporters, *pgp-1, pgp-5*, and *pgp-8*; and the hydrogen-myo-inositol symporters, *hmit-1.1* and *hmit-1.2* (Figure S4A). While lipid depletion and transcriptional fluctuations upon *hsf-1* RNAi resemble those of starvation conditions reported to activate NHR-49 and increase β-oxidation (Van Gilst et al., 2005b), food availability and intake were unaffected in *hsf-1* RNAi conditions, as indicated by comparable pharyngeal pumping rates and ingestion of fluorescent *Escherichia coli* (Figures S4B–S4D). Since *hsf-1* RNAi diminished Rab GTPase association with endocytic vesicles (Figure 3F), we hypothesized that impaired endocytic trafficking causes lipid depletion through malabsorption. Utilizing a *rab-11.2* null mutant worm strain, *rab-11.2(syb2999)*, we sought to determine whether NHR-49-dependent *rab-11.2* activation helps restore vesicular trafficking and lipid homeostasis in *hsf-1* RNAi conditions.

**Figure 4.**
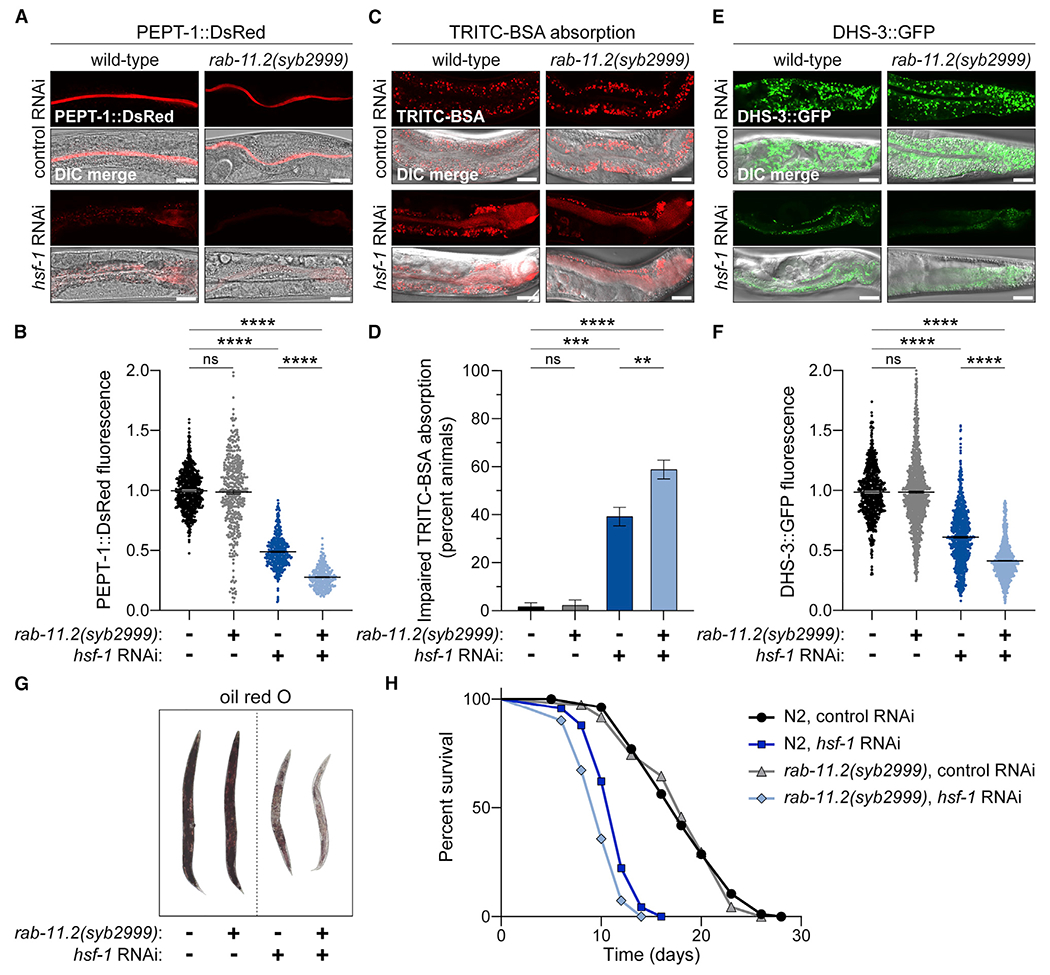
Adaptation to impaired apical recycling and malabsorption by *rab-11.2* activation (A–H) Comparative analyses of wild-type and *rab-11.2(syb2999)* mutant *C. elegans* treated with control or *hsf-1* RNAi. (A and B) Steady-state expression of apical peptide transporter, PEPT-1::DsRed. (A) Representative micrographs. Scale bar, 25 μm. (B) Relative fluorescence by flow cytometry. Mean ± SEM for, from left to right, 709, 479, 401, and 331 animals across three replicate experiments. ns, not significant; ****p < 0.0001 by two-way ANOVA with Tukey’s multiple comparisons. (C and D) TRITC-BSA absorption into the intestinal epithelium following its dietary supplementation. (C) Representative micrographs. Scale bar, 25 mm. (D) Percentage of animals that absorbed less than 50% of ingested TRITC-BSA into the intestinal epithelia. Mean ± SEM for three replicate experiments (n ≥ 118 animals per condition). **p = 0.0084, ***p = 0.0001, ****p < 0.0001 by two-way ANOVA with Tukey’s multiple comparisons. (E and F) Lipid droplet marker, DHS-3::GFP, expression. (E) Representative micrographs. Scale bar, 25 μm. (F) Relative fluorescence by flow cytometry. Mean ± SEM for (from left to right) 852; 1,095; 1,196; and 1,113 animals across three replicate experiments. ****p < 0.0001 by two-way ANOVA with Tukey’s multiple comparisons. (G) Representative micrographs of oil red O staining. (H) Lifespan curves for N2 (wild-type) and *rab-11.2(syb2999)* mutant *C. elegans*. Shown are representative lifespans of two replicates with approximately 100 animals per condition (see Table S3). p < 0.0001 by log rank test. See also Figure S4 and Table S3.

Examination of the proton-coupled dipeptide transporter, PEPT-1::DsRed, and the P-glycoprotein-related ABC transporter, PGP-3::mCherry, which reside at the apical intestine and facilitate transport across the digestive tract, revealed a dramatic reduction in their apical surface residency upon *hsf-1* RNAi (Figures 4A, 4B, S4E, and S4F). Transcript levels of *pept-1* and *pgp-3* remained unchanged upon *hsf-1* RNAi and therefore did not account for the dramatic losses in steady-state protein levels (Figure S4G). In *rab-11.2(syb2999)* mutant animals, we observed that preventing *rab-11.2* activation further exacerbated defects in the apical localization of PEPT-1::DsRed upon *hsf-1* RNAi (Figures 4A and 4B). These data suggest that increasing endocytic recycling via *rab-11.2* activation helps maintain surface residency and steady-state levels of apical proteins in conditions of impaired vesicular networks.

To examine how loss of apical transporters by *hsf-1* RNAi affects cellular absorption of environmental resources, we monitored the epithelial internalization of fluorescent labeled bovine serum albumin (BSA), TRITC-BSA, and the styryl dye, FM4-64, across the intestinal lumen. In both cases, absorption was reduced in animals treated with *hsf-1* RNAi (Figures 4C, 4D, and S4H). Furthermore, malabsorption upon *hsf-1* RNAi was worsened in *rab-11.2(syb2999)* mutants (Figures 4C, 4D, and S4H). Consistent with these observations, lipid depletion upon *hsf-1* RNAi was also exacerbated in *rab-11.2(syb2999)* mutants, as shown by reduced DHS-3::GFP fluorescence and oil red O staining (Figures 4E–4G, and S4I). Malabsorption and lipid depletion upon *hsf-1* RNAi likely result from a loss of bulk transporters and transport machinery at the apical intestine.

NHR-49-mediated activation of *rab-11.2* transcription appears to be an adaptive mechanism to improve cellular absorption and increase lipid deposition. Since *hsf-1* expression and activity declines with age (Ben-Zvi et al., 2009; Egge et al., 2019), we sought to examine how induction of *rab-11.2* transcription upon *hsf-1* RNAi affects age determination. The average lifespan of *rab-11.2(syb2999)* mutant animals was comparable with wild-type N2 animals (Figure 4H; Table S3), suggesting that basal *rab-11.2* expression under permissive conditions is dispensable for normal aging. However, preventing *rab-11.2* activation via the *rab-11.2(syb2999)* mutation under stressful conditions caused by *hsf-1* RNAi further shortened lifespan (Figure 4H; Table S3). Thus, NHR-49-dependent activation of *rab-11.2* plays an important role in cellular and physiological adaptability to metabolic stress caused by loss of *hsf-1*.

### Enteric actin destabilization and aggregation by loss of *hsf-1* impairs Rab GTPase homeostasis

**Figure 5.**
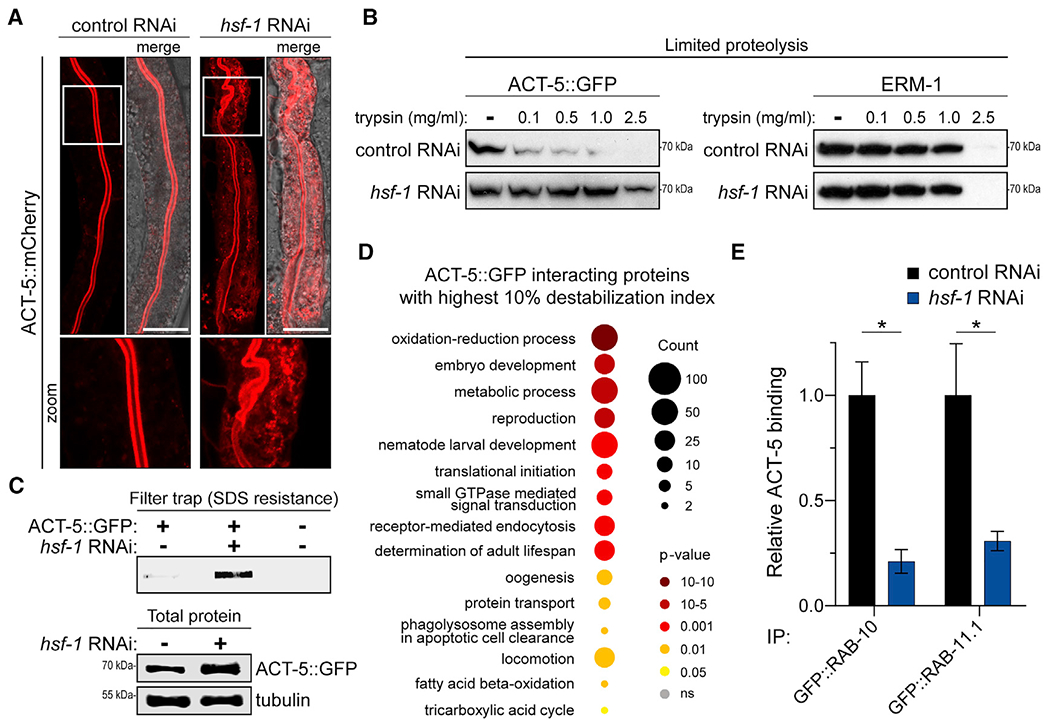
Enteric actin destabilization and aggregation by *hsf-1* RNAi impairs its interactions with endocytic machinery (A) Representative micrographs of intestinal actin variant, ACT-5::mCherry, localization. Scale bar, 25 μm. (B) Limited proteolysis of enteric ACT-5::GFP and cytoskeletal anchor protein, ERM-1. Lysates from transgenic *C. elegans* in control or *hsf-1* RNAi conditions were treated with increasing concentrations of trypsin and resolved by western blot. See Figures S5A and S5B for quantification of proteolysis. (C) Filter trap analysis of ACT-5::GFP SDS resistance, top, and western blot analysis of total ACT-5::GFP with tubulin loading control, bottom, from transgenic worms treated with control or *hsf-1* RNAi. Shown is a representative blot from three replicate experiments. (D) GO term analysis of the ACT-5 interacting proteins with the highest (top 10%) destabilization index values (relative change in solubility ratio upon *hsf-1* RNAi). Displayed are the most over-represented biological processes. p value represented by color; number of proteins (count) represented by circle size. See Table S4 for protein list. (E) Relative ACT-5 enrichment in GFP::RAB-10 and GFP::RAB-11.1 co-immunoprecipitations, detected by LC-MS/MS. Mean ± SEM for two biological replicates. *p = 0.0172 and 0.0140 by two-way ANOVA with Tukey’s multiple comparisons. See also Figure S5 and Table S4.

While our data demonstrate that HSF-1 affects the nucleocytoplasmic dynamics of NHR-49, it remained unclear how loss of *hsf-1* impairs Rab GTPase homeostasis. Previous studies identified an enteric actin isoform, ACT-5, whose hyperphosphorylation by the JUN kinase, KGB-1, upon *hsf-1* RNAi promotes its subcellular redistribution away from the subapical terminal web of the digestive tract (Egge et al., 2019). The actin network is intricately interwoven with cellular metabolism, and links between cytoskeletal dynamics and energy homeostasis are beginning to emerge (Hu et al., 2016). Actin has recently been shown to influence glycolysis (Park et al., 2020), but it remains unclear whether the actin network can affect lipid metabolism. Since Rab GTPases mediate vesicular trafficking along the actin network, we sought to determine whether actin network defects caused by *hsf-1* RNAi might affect Rab GTPase dynamics. Consistent with previous studies (Egge et al., 2019), *hsf-1* RNAi caused atypical ACT-5::mCherry accumulation in the intestines of adult animals (Figure 5A). Biochemical characterization revealed that ACT-5::GFP exhibited increased resistance to protease treatment upon *hsf-1* RNAi (Figures 5B and S5A). This appears to be specific for ACT-5, as *hsf-1* RNAi did not alter proteolysis of the cytoskeletal anchor protein, ERM-1 (Figures 5B and S5B). Ultracentrifugation and filter trap analysis of worm extracts showed elevated ACT-5::GFP in insoluble fractions and increased resistance to SDS upon *hsf-1* RNAi (Figures 5C and S5C). The biochemical properties of this insoluble ACT-5::GFP material, including its resistance to protease digestion and SDS treatment despite the absence of ATP, are not consistent with those reported for filamentous actin (Dancker and Fischer, 1989) and likely represent alternate, non-productive ACT-5 aggregates.

Aberrant assembly of proteins into macromolecular structures can physically sequester and inactivate associated proteins (Olzscha et al., 2011). Thus, we sought to determine whether aberrant ACT-5::GFP aggregates formed upon *hsf-1* RNAi might act in a similar fashion. ACT-5::GFP was immunoprecipitated from soluble and insoluble protein fractions isolated via ultracentrifugation and then analyzed by LC-MS/MS. A destabilization index was assigned to rank ACT-5 interacting proteins based on their shift from soluble to insoluble fractions upon *hsf-1* RNAi. GO term analysis of the top 10% of destabilized proteins identified receptor-mediated endocytosis (GO: 0007264) and small GTPase-mediated signal transduction (GO: 0006898) among the most enriched biological terms (Figure 5D; Table S4). This suggests that ACT-5 aggregation promotes sequestration and inactivation of small G proteins, including Rab GTPases and other endocytic regulators. In support, ACT-5 exhibited decreased binding to both GFP::RAB-11.1 and GFP::RAB-10 in *hsf-1* RNAi conditions, as detected by LC-MS/MS analysis of GFP::RAB-10/11.1 immunoprecipitations (Figure 5E). Thus, in addition to driving intestinal barrier dysfunction (Egge et al., 2019), disruption of the enteric actin network by *hsf-1* RNAi likely impairs endosomal trafficking.

### Disrupting RAB-11.1 interaction with actin is sufficient to activate the intracellular lipid surveillance response

**Figure 6.**
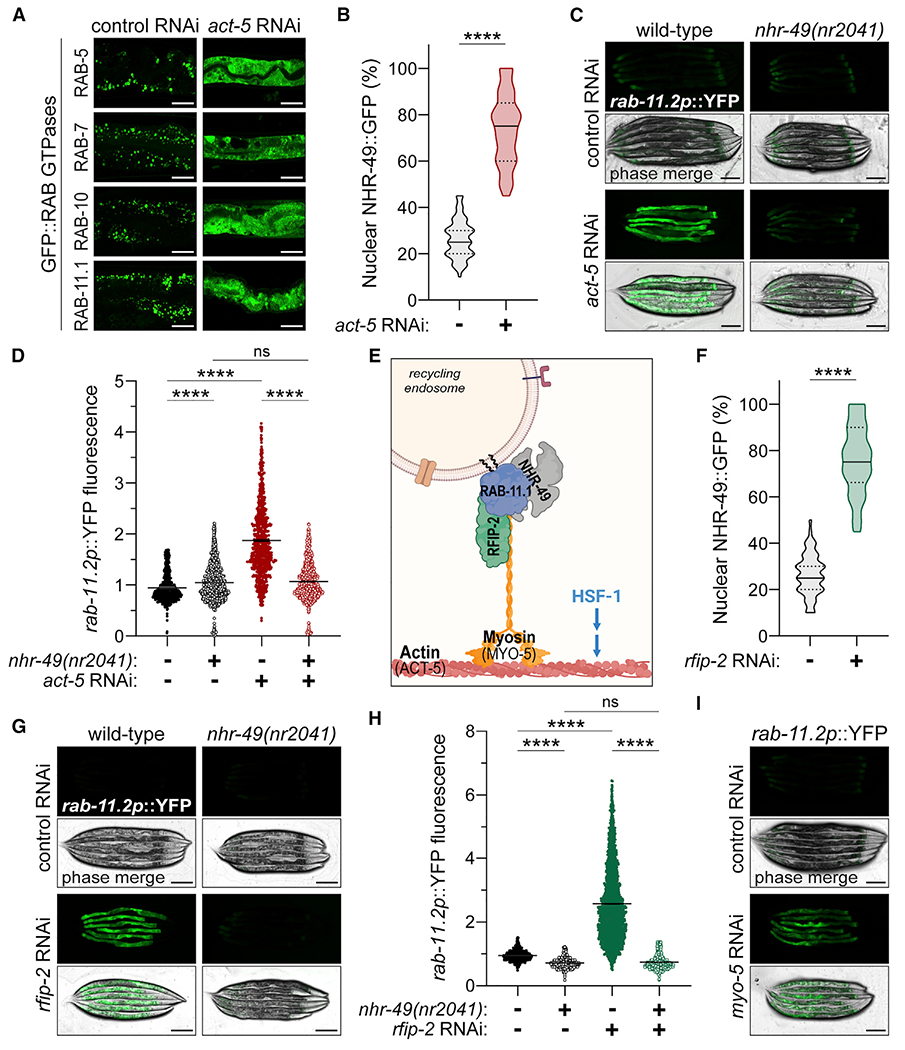
Disrupting vesicular binding to actin filaments is sufficient to activate the intracellular lipid surveillance response (A) Representative micrographs of intestinal GFP::RAB transgenes in adult *C. elegans* treated with control or *act-5* RNAi. Scale bar, 25 μm. (B) Percentage of intestinal epithelia with nuclear-enriched NHR-49::GFP. Median with quartiles for 60 animals per condition across three replicate experiments. ****p < 0.0001 by two-tailed unpaired t test. (C and D) Fluorescence of the *rab-11.2p*::YFP transcriptional reporter in wild-type and *nhr-49(nr2041)* mutant *C. elegans* on control or *act-5* RNAi. (C) Fluorescence micrographs. Scale bar, 200 μm. (D) Relative fluorescence by flow cytometry. Mean ± SEM for, from left to right, 940, 845, 1,504, and 845 animals across three replicate experiments. ****p < 0.0001 by two-tailed unpaired t test. (E) Schematic representing protein interactions that tether RAB-11.1-positive vesicles to enteric actin cytoskeleton. (F) Percentage of intestinal epithelia with nuclear-enriched NHR-49::GFP in adult *C. elegans* treated with control or *rfip-2* RNAi. Median with quartiles for 60 animals per condition across three replicate experiments. ****p < 0.0001 by one-way ANOVA with Sidak’s multiple comparisons. (G and H) Fluorescence of the *rab-11.2p*::YFP transcriptional reporter in wild-type and *nhr-49(nr2041)* mutant *C. elegans* on control or *rfip-2* RNAi. (G) Fluorescence micrographs. Scale bar, 200 μm. (H) Relative fluorescence by flow cytometry. Mean ± SEM for, from left to right, 3,322, 3,612, 2,606, and 3,692 animals across three replicate experiments. ****p < 0.0001 by one-way ANOVA with Sidak’s multiple comparisons. (I) Fluorescence micrographs of the *rab-11.2p*::YFP transcriptional reporter on control and *myo-5* RNAi. Scale bar, 200 μm. See also Figure S6.

Since actin dynamics are essential for endocytosis and cellular uptake of nutrients (Kaksonen et al., 2003; Mooren et al., 2012), we investigated whether destabilization and aggregation of the enteric actin network by *hsf-1* RNAi disrupts Rab-mediated vesicular transport and cellular absorption. Similar to *hsf-1* RNAi, impairing the enteric actin network via *act-5* RNAi disrupted the subcellular distribution of all GFP::RAB proteins examined and impaired absorption of both TRITC-BSA and FM4-64 into the intestinal epithelia (Figures 6A, S6A, and S6B). Fluorescence of the lipid droplet marker, DHS-3::GFP, was significantly reduced upon *act-5* RNAi, and targeted lipidomics confirmed corresponding geranylgeranyl depletion (Figures S6C–S6E). In accordance, *act-5* RNAi was sufficient to promote NHR-49::GFP nuclear accumulation, activate the *acs-2p*::GFP transcriptional reporter, and induce NHR-49-dependent fluorescence of the *rab-11.2p*::YFP transcriptional reporter (Figures 6B–6D, S6F, and S6G). Therefore, disruption of Rab GTPase dynamics via loss of *hsf-1* and its ability to maintain the integrity of the enteric actin network coincides with the activation of the intracellular lipid surveillance response.

Disruption of the enteric actin network yields pleotropic effects on cellular function, so we utilized a more precise means of disrupting interactions between the actin network and RAB-11.1, the critical determinant of NHR-49 cytosolic sequestration to endocytic vesicles (Watterson et al., 2022). The Rab11 family interacting proteins (Rab11FIPs) directly couple Rab11 to cytoskeletal transport machinery (Baetz and Goldenring, 2013) (Figure 6E), providing an alternate genetic approach to investigate the consequences of impairing ACT-5/RAB-11.1 interactions. While mammals possess five Rab11FIPs, *C. elegans* possesses two homologs, RFIP-1 and RFIP-2, of which RFIP-2 is most closely homologous to the myosin-interacting Rab11FIP2 (Baetz and Goldenring, 2013). We confirmed that reduced expression of *rfip-2* by RNAi disrupted GFP::RAB-11.1 association with vesicles (Figure S6H), which consequently diminished PEPT-1::DsRed levels and impaired absorption of TRITC-BSA and FM4-64 (Figures S6I–S6K). Importantly, *rfip-2* RNAi promoted NHR-49::GFP nuclear accumulation as well as *acs-2* and *rab-11.2* transcriptional activation (Figures 6F–6H, S6L, and S6M). Yet *rfip-2* RNAi did not reduce DHS-3::GFP lipid droplet marker fluorescence (Figures S6N and S6O), suggesting RFIP-2 may help facilitate the RAB-11.1/NHR-49 interaction. To further validate our proposed mechanism of lipid surveillance activation via impaired ACT-5/RAB-11.1 interaction, we tested RNAi for various muscle and non-muscle myosin motors, which tether and transport endocytic vesicles along the actin cytoskeleton. Of those examined, *myo-5* RNAi, and to a lesser extent *myo-1* RNAi, promoted NHR-49::GFP nuclear accumulation and *rab-11.2p*::YFP activation (Figures 6I, S6P, and S6Q). These data demonstrate that genetically impairing transport of Rab11 endocytic vesicles along the actin cytoskeletal network is sufficient to activate the lipid surveillance pathway through NHR-49. While actin misfolding is likely not the only means by which loss of *hsf-1* affects lipid surveillance, the data presented in this study suggest that, through its ability to stabilize the enteric actin network, HSF-1 integrates lipid metabolism, absorption, and aging via the NHR-49-mediated intracellular lipid surveillance pathway.

## DISCUSSION

Ancient regulators of transcription and cellular adaptation have the capacity to expand their functional roles through the evolutionary emergence of new molecules. The ancestral role of HSF1 as a mediator of protein folding and the heat shock response has persisted throughout evolution (Baird et al., 2014). However, we are beginning to appreciate its integration into other essential cellular processes, many of which have never been reported in unicellular systems, including development, lipid metabolism, and age determination (Akerfelt et al., 2010). Our study identified the nuclear hormone receptor, NHR-49, as being required for HSF-1 to affect lipid metabolism and age regulation but not thermotolerance. While HSF1 is present in unicellular organisms, nuclear hormone receptors exist only in multicellular organisms (Escriva et al., 1998; Sladek, 2011). Our findings suggest that the evolutionary emergence of NHR-49 enabled HSF-1 to adopt new physiological roles while still retaining its ancient function in thermal adaptation. This is supported by lipid droplet staining in *S. cerevisiae*, in which reduced *hsf-1* expression did not affect lipid droplet levels. However, a more comprehensive study across several unicellular organisms is needed to solidify the hypothesis that HSF1 has no role in lipid deposition in unicellular organisms.

The roles of HSF-1 and the metazoan-specific NHR-49 converge on Rab GTPase-mediated endosomal trafficking and lipid metabolism, linking cellular absorption with metabolic demand. Loss of *hsf-1* activates NHR-49-mediated transcription of genes involved β-oxidation as well as cellular absorption such as *rab-11.2.* While we demonstrate NHR-49-dependent *rab-11.2* activation, it remains unclear whether *rab-11.2* is a direct transcriptional target of NHR-49. It is likely that other transcriptional regulators are also involved in *rab-11.2* activation, such as NHR-66 or NHR-80 (Pathare et al., 2012). As an established regulator of β-oxidation in *C. elegans* (Van Gilst et al., 2005a, 2005b), the role for NHR-49 in facilitating nutrient absorption through endocytic recycling may begin to explain its requirement for maintaining lipid reserves in adult worms (Ratnappan et al., 2014) (Figure 2). This seemingly opposing function for NHR-49 in lipid breakdown as well as maintenance highlights the growing complexity of its roles in regulating lipid homeostasis. Perhaps NHR-49 incurs versatile functionality through different co-factor interactions. In support, NHR-49 has been shown to regulate different subsets of genes depending on its dimerization with NHR-66 versus NHR-80 (Pathare et al., 2012). With respect to NHR-49 and HSF-1 transcriptional activity, innate immunity was the most enriched biological process by GSEA analysis of shared differentially expressed genes upon *hsf-1* RNAi and *nhr-49(nr2041)* (Figure 1G). The emerging role for NHR-49 in mediating immune response to infection is being characterized (Naim et al., 2021), and further studies will better elucidate how lipid surveillance affects immunity.

We implicate Rab GTPases as the central component in linking HSF-1-mediated stabilization of the enteric actin network with NHR-49 nucleocytoplasmic dynamics and activity. Representing the largest family of small G proteins, Rab GTPases control intracellular vesicular transport (Stenmark, 2009). However, interestingly, *rab-11.2* is selectively activated by the lipid surveillance response. First, this presents an atypical mode of small G protein regulation, which typically occurs through posttranslational means such as nucleotide exchange and co-factor interactions (Stenmark, 2009). Second, this introduces the question as to why cells preferentially activate Rab11. While most Rab GTPases have specialized roles in varying processes of the endo-lysosomal system, there appears to be some promiscuity or moonlighting roles for the different GTPases. Rab11 has historically been ascribed as the master regulator of endocytic recycling, which is important for retaining residency of membrane proteins on the cell surface (Welz et al., 2014). However, several reports have also linked Rab11 function with Golgi-to-plasma membrane transport (Winter et al., 2012); Welz et al., 2014). Thus, if the cell seeks to restore residency of membrane proteins at the apical surface, Rab11 is well suited to perform this task. Third, it remains unclear why cells preferentially induce expression of *rab-11.2* over *rab-11.1.* We hypothesize that this Rab11 system may function in a similar manner to the Heat Shock Protein 70 (HSP70) chaperone system (Krakowiak et al., 2018). Cells possess a cognate enzyme, HSC70, which is abundant and performs protein folding functions in permissive conditions. When HSC70 becomes compromised or limited due to thermal stress or widespread protein misfolding, cells activate the functionally redundant inducible HSP70, which likely acts through mass action to refold the respective substrates (Qian et al., 2006). A previous study supports the possibility that the Rab11 system acts similarly, as preventing *rab-11.2* induction exacerbates all phenotypes observed upon *rab-11.1* RNAi (Watterson et al., 2022). Future studies will elucidate these outstanding questions regarding the transcriptional activation and cellular function of *rab-11.2.*

In a broader context, linking cytosolic protein homeostasis to intracellular lipid surveillance provides potential advantages under times of cellular stress. Should cytosolic protein homeostasis mechanisms fail or decay, as reported with age (Ben-Zvi et al., 2009), protein misfolding throughout the cell might become so widespread that it promotes malabsorption. We propose in this study that loss of *hsf-1* impairs Rab GTPase-mediated trafficking through JUN kinase-mediated hyperphosphorylation of actin (Egge et al., 2019), but the observed actin aggregation may be driven by additional means such as impairments in proteasomal degradation. While we highlight the importance of the enteric actin network, we hypothesize that misfolding of other critical proteins, such as nutrient transporters, endosomal trafficking components, and metabolic enzymes, also has the potential to affect lipid surveillance pathway activity. In this manner, the cell might possess additional sensors whose misfolding-induced loss of function activate the lipid surveillance response. Nonetheless, increasing uptake and metabolism of extrinsic resources enables some means for cells to meet immediate energetic demands as well as elevate geranylgeranyl production to ensure continued resource uptake.

While endocytic recycling plays a critical role in nutrient absorption by maintaining transporters at the apical surface of intestinal epithelia, this molecular mechanism has the potential to extend to other biological paradigms that are affected by membrane protein abundance. This study examines two apical transporters in the intestine, but this HSF-1-mediated mechanism could be applied to a vast array of cell signaling paradigms. Promoting membrane recycling via activation of the intracellular NHR-49-dependent lipid surveillance response could, for example, improve antigen presentation for immune clearance, strengthen synaptic transmission in neurons, or prevent age-associated loss in tissue integrity caused by loss of cell junctions. Alternatively, suppression of lipid surveillance might serve to mitigate the spread of a viral infection by reducing the intake or secretion of viral particles from infected cells. Overall, the findings and questions presented in this study introduce potential avenues for further investigation of how protein folding and actin dynamics affect lipid sensing and metabolism as well as how inducible activation of endocytic recycling could be applied translationally.

### Limitations of the study

Our study was limited by the lack of complete genetic knockouts such as an *hsf-1* null mutation, which prevented us from performing epistasis experiments. Similarly, we were unable to isolate fertile *nhr-49(nr2041); hsf-1* OE animals from genetic crosses due to synthetic sterility. To overcome these limitations, we utilized RNAi combinations, which led to a range of phenotypes. Additionally, the *nhr-49(nr2041)* mutation may be an incomplete loss-of-function mutation, as *nhr-49(nr2041)* and *nhr-49* RNAi did not always yield the same phenotypes. Moreover, quantification of geranylgeranyl levels via targeted lipidomics is not trivial and required specialized equipment, expensive reagents, and specific expertise. This limited the number of experimental conditions and time points in which we could measure geranylgeranyl levels. Furthermore, LC-MS/MS analysis does not distinguish between direct and indirect interactions in the GFP::RAB or GFP::ACT-5 immunoprecipitations. We would have liked to demonstrate direct physical interactions such as RFIP-2 with cytoskeletal machinery or endocytic components but were limited by the lack of available antibodies. Similarly, we lacked the tools to determine the nature of NHR-49 interaction with endocytic components like RAB-11.1 and RFIP-2.

## STAR★METHODS

### RESOURCE AVAILABILITY

#### Lead contact

Further information and requests for resources and reagents should be directed to and will be fulfilled by the lead contact, Peter Douglas (peter.douglas@utsouthwestern.edu).

#### Materials availability

Worm strains generated in this study are available upon request from the lead contact.

#### Data and code availability

- All data generated and analyzed during this study are included in this article and its supplemental information. Transcriptomic data files that support the findings of this study in *C. elegans* have been deposited in the NCBI Gene Expression Omnibus (GEO) under accession GSE199971.
- The paper does not report original code.
- Any additional information required to reanalyze the data reported in this paper is available from the lead contact upon request.

### EXPERIMENTAL MODEL AND SUBJECT DETAILS

#### C. elegans *strains and maintenance*

Worm strains were maintained at 15°C on an OP50 *E. coli* lawn grown on standard NGM plates. For experimental purposes, strains were grown on NGM plates supplemented with HT115 *E. coli* at 20°C unless otherwise noted. Age-synchronization was performed by hypochlorite treatment of gravid animals to obtain eggs. AGP33a (*glmEx8(nhr-49p::nhr-49::gfp* + *myo3p::mCherry); nhr-49(nr2041) I*) worms were obtained from the laboratory of Dr. Arjumand Ghazi (Ratnappan et al., 2014). MZE1 (*unc-119(ed3) III; cbgIs91[pept-1p::pept-1::DsRed* + *unc-119(*+*)]; rrf-3(pk1426) II; pwls69[vha-6p::gfp::rab-11* + *unc-119(*+*)]*) worms were obtained from the laboratory of Dr. Marino Zerial (Winter et al., 2012). ERT137 (*jyIs17[act-5p::mCherry::act-5* + *ttx-3p::rfp] IV; dkIs8[chc-1p::chc-1::gfp]*) worms were obtained from the laboratory of Emily Toemel (Szumowski et al., 2016).

The following strains were obtained from the Caenorhabditis Genetics Center: N2 (ancestral wild-type), CF512 (*rrf-3(b26) II; fem-1(hc17) IV*), AGD710 (*uthIs235[sur5-p::hsf-1* + *myo-2p::tdTomato]*), STE68 (*nhr-49(nr2041) I*), LIU1 (*ldrIs1[dhs-3p::dhs-3::gfp* + *unc-76(*+*)]*), WBM170 (*wbmEx57[acs-2p::gfp* + *rol-6(su1006)]*), RT327 (*pwIs72[vha-6p::gfp::rab-5* + *Cbr-unc-119(*+*)] II*), CL2070 (*dvln70[hsp-16.2p::gfp* + *rol-6(su1006)]*), RT476 (*pwIs170[vha6p::gfp::rab-7* + *Cbr-unc-119(*+*)]*), RT525 (*pwIs206[vha6p::gfp::rab-10* + *Cbr-unc-119(*+*)]*), RT311 (*pwls69[vha-6p::gfp::rab-11.1* + *unc-119(*+*)]*) and OG472 (*drSi2[vha-6p::3xflag::pgp-3/CFTR(wt)::mCherry] II*).

PMD19 (*jyIs13x3[gfp::act-5] II; fer-15(b26) II; fem-1(hc17ts) IV*) (Egge et al., 2019) as well as PHX2999 (*rab-11.2(syb2999) I*), PMD118 (*utsEx14[rab-11.2p::yfp]; nhr-49(nr2041) I*), PMD124 (*utsIs3[rab-11.2p::yfp]*), PMD90 (*fem-1(hc17ts) IV; pwls209[vha-6p::gfp::rab-11.1* + *Cbr-unc-119(*+*)]*), PMD91 (*fem-1(hc17ts) IV; pwls69[vha-6p::gfp::rab-10* + *Cbr-unc-119(*+*)]*), PMD150 (*utsIs4 [glmEx8(nhr-49p::nhr-49::GFP* + *myo3p::mCherry]; nhr-49(nr2041) I*), PMD169 (*cbgIs91[pept-1p::pept-1::DsRed* + *unc-119(+)]; rrf-3(pk1426) II; rab-11.2(syb2999) I*) and PMD170 (*ldrIs1[dhs-3p::dhs-3::gfp + unc-76(*+*)];rab-11.2(syb2999) I*) were described previously (Watterson et al., 2022).

The following strains were produced for this study: PMD17 (*pwls69[vha-6p::gfp::rab-11* + *unc-119(*+*)]; jyIs17[act-5p::mCherry::act-5* + *ttx-3p::rfp] IV*) was made by crossing RT311 to ERT137, PMD121 (*N2; wild-type nhr-49*) and PMD122 (*N2; nhr-49(nr2041) I*) by crossing N2 to STE68, of which both wild-type and *nhr-49(nr2041)* mutant homozygotes from the F2 generation were maintained, PMD142 (*ldrIs1[dhs-3p::dhs-3::gfp* + *unc-76(+)]; nhr-49(nr2041) I*) by crossing LIU1 to STE68, PMD157 (*ldrIs1[dhs-3p::dhs-3::gfp* + *unc-76(*+*)]; uthIs235[sur5-p::hsf-1* + *myo-2p::tdTomato]*) by crossing LIU1 to AGD710, and PMD168 (*wbmEx57[acs-2p::gfp* + *rol-6(su1006)]; uthIs235[sur5-p::hsf-1* + *myo-2p::tdTomato]*) by crossing WBM170 to AGD710. See Table S5 for worm strains associated with figure panels.

#### S. cerevisiae *strains and maintenance*

*S. cerevisiae* strains were maintained on yeast extract peptone dextrose (YEPD) agar plates at 4°C. Growth media was supplemented with 200 μg/μL G418 to select for the *hsf1*-DAmP strain (Breslow et al., 2008). Yeast were cultured overnight at 30°C with vigorous shaking. Liquid cultures were diluted the following morning to OD_600_ ~ 0.4 and allowed to re-enter log phase of growth for at least 4 h prior to growth rate assessment and lipid droplet staining. Note: the strains exhibited comparable growth rates.

### METHOD DETAILS

#### Single worm genotyping

To identify animals carrying the *rab-11.2(syb2999)* or *nhr-49(nr2041)* mutations, individual worms from the F2 generation of genetic crosses were transferred to NGM plates seeded with OP50 and allowed to lay eggs. The F2 adult worms were then frozen in 10 μL of worm genotyping lysis buffer [10 mM Tris-Base (pH 8.2), 50 mM KCl, 2.5 mM MgCl_2_, 0.45% Tween-20, and 60 μg/mL Proteinase K (VWR), added immediately before use] for at least 15 min at −80°C. Worms were lysed for 1 h at 60°C, followed by Proteinase K inactivation for 15 min at 95°C. A standard PCR reaction was performed using primers which bound immediately upstream and downstream of the respective mutation. To identify worms carrying the *rab-11.2(syb2999)* and *nhr-49(nr2041)* mutations, PCR products were loaded onto a 1.0% agarose/TBE gel and electrophoresis was performed at 100 V for 1 h. Mutants were identified according to PCR product size [1,000 bp (*nhr-49* wild-type) vs 100 bp (*nhr-49(nr2041)*) and 986 bp (*rab-11.2* wild-type) vs 230 bp (*rab-11.2(syb2999)*)].

#### RNAi administration

Interfering RNAs were obtained from either the Vidal or Ahringer RNAi libraries. All RNAi experiments were performed using HT115 *E. coli* containing either L4440 empty vector control or various RNAi constructs. Liquid cultures of RNAi were grown in Terrific Broth (TB)for 14 h at 37°C and then induced with 1 mM IPTG for 4 h at 37°C. Cultures were concentrated to 1/10 of their original volume via centrifugation at 3,000 x g for 10 min, seeded onto RNAi plates (60 mm or 100 mm, containing 0.1 mg/mL carbenicillin and 1 mM IPTG, added 24 h prior to seeding), and allowed to dry for 1-2 days at room temperature, protected from light. For *act-5* RNAi experiments, worms were treated with a 5%:95% *act-5*:control RNAi mixture due to developmental defects caused by 100% *act-5* RNAi.

For experimental analyses of transgene fluorescence, transgenic worm strains expressing NHR-49::GFP or the *hsp-16.2p::GFP, acs-2p*::GFP and *rab-11.2p*::YFP transcriptional reporters were cultured on RNAi until day 1 of adulthood. Transgenic worms expressing GFP::RAB constructs were cultured on RNAi until day 2 of adulthood. Transgenic worms expressing PGP-3::mCherry or PEPT-1::DsRed were cultured on RNAi until day 3 or 4 of adulthood, respectively. Transgenic worms expressing DHS-3::GFP were cultured on RNAi until day 2 of adulthood for experiments with *act-5* RNAi, day 3 of adulthood for wild-type versus *rab-11.2(syb2999)* mutant comparisons and experiments with *rfip-2* RNAi, or day 5 of adulthood for all other experiments. Transgenic worms expressing ACT-5::mCherry were cultured on RNAi until day 3 of adulthood. Specific RNAi culture conditions for all other worm strains and experiments are described in the respective methods sections.

#### Ultrastructural analysis and lipid density

Transmission electron microscopy was performed as previously described (Egge et al., 2019). In brief, day 5 adult *C. elegans* were fixed in 2.5% glutaraldehyde, 1% paraformaldehyde in 0.05M sodium cacodylate buffer (pH 7.4) plus 3.0% sucrose overnight at 4°C, rinsed in 0.1M cacodylate buffer, embedded in 3% agarose, and sliced into small blocks (1 mm^3^). Blocks were rinsed (0.1M cacodylate buffer), post-fixed in 1% osmium tetroxide for 2 h at room temperature, rinsed with buffer followed by double distilled water, and *en bloc* stained with 2% aqueous uranyl acetate for 1 h at room temperature. Next, blocks were dehydrated with increasing concentrations of ethanol, transitioned into Spurr’s resin with propylene oxide, infiltrated with Spurr’s resin, and polymerized in a 70°C oven overnight. Blocks were sectioned with a diamond knife (Diatome) on a Leica Ultracut 7 ultramicrotome (Leica Microsystems), transferred to copper grids, and post-stained with 2% aqueous uranyl acetate and lead citrate. Images were acquired on a Tecnai G^2^ spirit transmission electron microscope (Thermo Fisher) equipped with a LaB_6_ source using a voltage of 120 kV. For lipid droplet density threshold analysis, TEM micrographs were inverted and converted to black & white with maximum black saturation using Adobe Photoshop. Relative lipid droplet density was measured from histogram quantification of black threshold (Adobe Photoshop). Ten micrographs were analyzed per condition. Threshold values were normalized to the mean of control RNAi and Prism 9 software (GraphPad) was used for statistical analysis.

#### Yeast monodansylpentane staining

To characterize lipid droplet levels in *S. cerevisiae*, cells were stained at various growth stages with a 1:1000 dilution of AUDODOT visualization dye (Abcepta SM1000a) for 15 min. Cells were then washed with distilled water, concentrated, and spotted directly onto microscope slides for imaging. The remaining cells were transferred to a flat-bottom 96-well plate and fluorescence (405_Ex_/450_Em_) was measured using a CLARIOstar plus fluorimeter (BMG Labtech). Background autofluorescence values determined from unstained yeast cells were subtracted from fluorescence values for each sample. Fluorescence readouts were normalized relative to wild-type control and Prism 9 software was used for statistical analysis.

#### oil red O staining and density quantification

To assess major fat stores, day 5 adult worms were fixed and stained with 60% oil red O Isopropanol solution as previously described (Watterson et al., 2022). In brief, worms were rinsed off plates, washed three times in 1x PBS, and then incubated while rocking in 2x MRWB [3.7% formaldehyde, 160 mM KCl, 40 mM NaCl, 14 mM EDTA, 0.2% β-mercaptoethanol] diluted to 1x in PBS for 1 h at room temperature. Following three washes with 1x PBS, worms were incubated in 60% isopropanol for 15 min and then stained with 60% oil red O for 18 h. Dye was then removed and replaced with 1x PBS + 0.01% Triton-X for 2 min, followed by three washes with 1x PBS. Worms were mounted on a 1% agarose pad for imaging. All experiments were replicated at least three times. For quantification of oil red O staining density, mean brightness from representative regions within the worm midsections was measured using ImageJ (brighter signal = less staining density). Stain brightness was measured from numerous worm micrographs acquired across three independent trials, and relative staining density was calculated by dividing the inverse of the measured values by the mean of the control for each replicate experiment.

#### Absorption assays

Assessment of TRITC-BSA and FM4-64 absorption efficiency was performed as previously described (Watterson et al., 2022). In brief, approximately 100 day 5 (*hsf-1* RNAi), day 3 (*rfip-2* RNAi), or day 1 (*act*-5 RNAi) adult worms were washed three times in M9 buffer and then incubated in 50 μL of 15 mM TRITC-BSA (Thermo Fisher Scientific A23016) or 0.4 mM FM4-64 (Thermo Fisher Scientific T3166) in an Eppendorf Thermomixer C shaker set to 300 rpm for 2 h at 20°C. Worms were then washed three times with M9 buffer and allowed to recover on NGM plates seeded with OP50 for 2 h in the dark. For quantification of endocytosis efficiency, worms were visualized under an Axio Observer inverted microscope (Carl Zeiss Microscopy) at 50x magnification and scored according to absorption efficiency; absorption was considered impaired if less than 50% of TRITC-BSA/FM4-64 was absorbed across the intestinal epithelia. For imaging, worms were rinsed off plates with M9, centrifuged (1,000 x g) for 30 s, and mounted on slides in M9 supplemented with 100 mM levamisole.

#### Ingestion and pharyngeal pumping assays

For pharyngeal pumping rate and fluorescent *E. coli* ingestion analyses, age-synchronized worms were cultured on RNAi until day 1 of adulthood. The number of pharyngeal pumps per min were counted for 10 randomly selected worms per condition for each replicate experiment using a Leica S8AP0 light microscope. Worms were then rinsed off plates with M9 buffer, centrifuged (1,000 x g) for 30 s, and transferred to RNAi plates seeded with a mixture of HT115 *E. coli* expressing RNAi (90%) and HT115 *E. coli* expressing mCherry (10%) and incubated for 2 h in the dark. Immediately following incubation, worms were rinsed off plates, washed three times in M9 buffer, and mCherry fluorescence within the intestinal lumen was measured by large-particle flow cytometry. Alignment of fluorescence profiles by time-of-flight (TOF) were performed automatically by FlowPilot (ver. 2.6.1, Union Biometrica) software.

#### Nuclear NHR-49 quantification

Assessment of nuclear NHR-49::GFP was performed as previously described (Watterson et al., 2022). Age-synchronized worms on HT115 *E. coli* expressing RNAi were visualized under an Axio Observer inverted microscope (Carl Zeiss Microscopy) at 100x magnification. Observers were blinded to sample condition by randomly assigning letters to the plates prior to visualization. The number of fluorescent intestinal nuclei were counted for each individual worm (at least 15 worms per condition for each biological replicate); NHR-49::GFP was considered nuclear if the fluorescence intensity in the nucleus exceeded that of the cytosolic signal proximal to the nucleus by at least 2-fold. Percentage was calculated by dividing the respective number of NHR-49::GFP-positive nuclei by 20 (total number of *C. elegans* intestinal cells). Percentage distributions for each condition were analyzed using Prism 9.

#### Microscopy

For fluorescence micrographs of transcriptional reporter worm strains, six randomly selected worms were aligned on NGM/Carb plates in a drop of M9 supplemented with 100 mM levamisole. Imaging was performed on a Zeiss Axio Observer inverted microscope using a Zeiss 20x Plan-Apochromat air immersion objective (0.8 NA, 0.55 mm WD) set to 80x magnification. Images were acquired using transmitted light and standard filter settings for excitation and emission of fluorescence probes and recorded on a CCD camera (Zeiss AxioCam MRm). Zeiss ZEN software was used to control acquisition. Exposure settings and additional processing parameters remained consistent among samples in each experiment. oil red O staining was observed using a Zeiss Axio Observer Z1 inverted microscope with an MRC-Color camera. Zeiss ZEN software was used to control acquisition. Confocal micrographs were acquired using a Leica SP8 confocal microscope (Leica, Buffalo Grove, IL) and Leica Application Suite X (LAS X) software. For *S. cerevisiae*, 1 μL of cultures were mounted on slides and imaged at 63x with oil immersion. For *C. elegans*, live worms were mounted in M9 supplemented with 100 mM levamisole and imaged at 40x or 63x with oil immersion. All images were acquired in xyz acquisition mode with line averaging. Lasers 405, 488 or 552 were used to excite fluorophores using hybrid detectors (HyD). Laser power, range, and gain were adjusted according to strain/experiment but remained consistent between replicates.

#### Large-particle flow cytometry

Age-synchronized worms were analyzed by flow cytometry through use of a COPAS FP-250 flow cytometer (Union Biometrica, Holliston, MA). Samples were acquired from a 96-well plate by an LP Sampler (Union Biometrica). Sample solution was comprised of M9 while flow sheath solution contained a proprietary recipe, COPAS GP Sheath Reagent (PN: 300-5070-100, Union Biometrica). Flow data was collected and processed using the FlowPilot software. Further data processing was performed in Excel (Microsoft) and statistical analysis in Prism 9. Extinction was detected using the 488 nm laser line with a 1.3 ND filter and gain of 1.0. DsRed and mCherry fluorophores were excited using a 561 nm laser; YFP and GFP were excited using a 488 nm laser. Gains for fluorescence detection were set to 2.0, and PMT voltage adjusted within the linear range of the instrument was consistent for each experiment (500 for YFP, mCherry, DsRed and 450 for GFP). For all analyses, fluorescence values represented the integral of signal intensity across the whole worm. Fluorescence values were normalized according to TOF to account for variation in worm size across RNAi conditions. For relative fluorescence of individual worm populations, fluorescence values were standardized relative to control. See Figure S7 for representative gating strategies.

#### Lifespan analysis

Lifespan experiments were conducted at 20°C as previously described (Egge et al., 2019). Lifespans were performed on worms fed OP50 *E. coli* or HT115 RNAi using the pre-fertile period of adulthood as day 0. Worms were transferred to fresh play every second day until day 12. Bagged, desiccated, or missing animals were censored from analysis. Prism 9 software was used for statistical analysis and significance was determined using the log-rank (Mantel-Cox) method.

#### Heat shock and thermotolerance assays

For analysis of *hsp-16.2p*::GFP transcriptional reporter activation by heat shock, day 1 adult worms grown at 20°C on HT115 *E. coli* containing RNAi were incubated at 33°C for 40 min, and then allowed to recover on OP50 *E. coli* for 4 h prior to imaging and analysis by large-particle flow cytometry. For analysis of thermotolerance, age-synchronized day 1 adult worms grown at 20°C on HT115 *E. coli* containing RNAi were placed at 34°C for 12 h as previously described (Baird et al., 2014). Following heat shock, worms were scored for viability. At least 80 worms were used per genotype and experiments were repeated at least three times. Prism 9 was used for statistical analysis.

#### Targeted lipidomics

Geranylgeranyl abundance was measured from lysates of day 1 adult CF512 worms cultured at 25°C as previously described (Watterson et al., 2022). In brief, a modified Bligh-Dyer extraction using equal parts H_2_O, methanol, and dichloromethane (DCM) was used to lyse cells and solubilize lipids from thawed worm pellets. The organic phase (containing lipids) was dried under nitrogen and samples were hydrolyzed with 0.5 M KOH to eliminate the abundant glycerolipids and glycerophospholipids. The organic layer was centrifuged, and the supernatant was transferred to a new glass insert and dried under nitrogen. Samples were resuspended in 450 μL 90% methanol, and 45 μL of each sample was injected into a Shimadzu LC-20XR HPLC and analysed on an SCIEX API 5000 mass spectrometer in positive electrospray mode. A 12 min liquid chromatography method was used with a binary gradient in which mobile phase A contained 90% methanol with 5 mM NH4OAc and phase B contained 100% methanol with 5 mM NH4OAc. Geranylgeranyl abundance was resolved using a Phenomenex Kinetex C18 column (100 × 3.0 mm, 1.7 μm particle size) held at 40°C. Total peak area reflecting the amount of geranylgeranyl in each sample was derived using Analyst Software (ver. 1.7, SCIEX).

#### RNA extraction and qPCR analysis

For quantitative PCR (qPCR), day 1 adult N2 worms cultured on HT115 *E. coli* containing RNAi were harvested in TRIzol (Thermo Fisher Scientific), flash frozen in liquid nitrogen, and stored at −80°C. RNA extraction and qPCR analysis were performed as previously described (Egge et al., 2021). In brief, frozen worm pellets were freeze-thawed three times, followed by a chloroform/isopropanol process for RNA extraction. RNA pellets were washed twice with ethanol and resuspended in 20 μL ddH_2_O. RNA quantification was performed using the DeNovix spectrometer (DS-11 FX+); 260/280 and 260/230 ratios were used to assess RNA quality. cDNA synthesis was conducted using the QuantiTect Reverse Transcription kit (Qiagen) according to manufacturer instructions. qPCR was performed with the iTaq^™^ Universal SYBR^®^ Green Supermix kit (Bio-Rad) and reactions were run using the CFX384 Real Time System (Bio-Rad). Primer sequences were designed to target genes of interest. For each gene, 3 independent biological repeats and 3 technical repeats were included in the analysis. Fidelity of PCR reactions was examined by melting curve analysis. The ΔC_t_ method was used to calculate the relative transcriptional abundance of the target genes and the two housekeeping genes, alpha tubulin (*tba-1*) and a putative iron-sulfur cluster assembly enzyme (*Y45F10D.4*). The geometric mean of the housekeeping genes was used to normalize target gene expression. ΔΔC_t_ represents the relative target gene abundance compared to control RNAi.

#### Illumina RNA sequencing

Three biological replicates of age-synchronized wild-type *nhr-49* (PMD121) and *nhr-49(nr2041)* (PMD122) worms cultured on control RNAi until late L4 larval stage were harvested in TRIzol (Thermo Fisher Scientific) and rapidly flash frozen in liquid nitrogen. RNA was purified by chloroform/phenol extraction followed by isopropanol precipitation and two washes with 75% ethanol before resuspension in 50 μL molecular biology grade water. Quality control, mRNA purification, and paired end 150 bp Illumina sequencing was performed by Novogene (Sacramento, CA) as previously described (Egge et al., 2019, 2021). Statistical analysis was performed using CLC Genomics Workbench software (version 9.5, Qiagen Bioinformatics, Aarhus, Denmark). Gene expression data were compared by Baggerly’s test with False Discovery Rate (FDR) correction. Genes were considered significantly regulated in *nhr-49(nr2041)* mutants when log_2_ fold change > ± 0.5 and *p*-value < 0.05.

#### Gene set comparison and enrichment analyses

RNA sequencing analysis of wild-type and *nhr-49(nr2041)* worms was performed for this study. RNA sequencing analysis of gene expression upon heat shock (33°C for 30 min) and/or *hsf-1* RNAi was previously performed (Brunquell et al., 2016) and downloaded from the NCBI BioProject repository (Accession PRJNA311958). Microarray datasets for wild-type and full length (FL) *hsf-1* overexpression were previously described (Baird et al., 2014). All analyses were performed in late larval stage L4 animals. CLC Genomics Workbench software was used to analyze statistical significance; gene expression data were compared by Baggerly’s test with False Discovery Rate (FDR) correction. Genes were considered significantly regulated when log_2_ fold change > ± 0.5 and *p*-value < 0.05 relative to the respective controls. The significantly regulated genes upon *hsf-1* RNAi and the *nhr-49(nr2041)* mutation were compared using the Gene List Venn Diagram resource from Source Forge. The DAVID Bioinformatics Resources 6.8 (Huang et al., 2009a; 2009b) was used for gene ontology (GO) term analysis of the shared significantly regulated genes, listed in Table S2. For analysis of fatty acid and lipid metabolism gene regulation upon *hsf-1* RNAi or *hsf-1* overexpression, all annotated genes involved in fatty acid and lipid metabolism or mobilization were identified using the GO Biological Function search tool in CLC software. Those differentially regulated upon *hsf-1* RNAi (*p*-value < 0.05) were cross-compared to the *hsf-1* overexpression microarray dataset. Relative expression values and associated statistics for these genes are represented in Table S1. Heat shock protein (*hsp*) expression upon heat shock (Brunquell et al., 2016) or the *nhr-49(nr2041)* mutation were compared relative to the respective controls. Heatmap generation and statistical analysis were performed using Prism 9 software.

#### ChIP-seq dataset analysis

Chromatin immunoprecipitation sequencing (ChIP-seq) for HSF-1 was performed in late L4 larvae treated with or without heat shock (33°C for 30 min) as previously described (Li et al., 2016). Datasets were obtained from GeoDatasets (GSE81521) and analyzed with CLC Genomics Workbench software to identify HSF-1 binding peaks across the *C. elegans* genome. Images were acquired for HSF-1 binding peaks in the 5′ untranslated regions (UTR) of genes of interest.

#### Proteomics sample preparation and LC-MS/MS

Age-synchronized transgenic worms were cultured on RNAi (NGM/carb/IPTG) plates supplemented with control or *hsf-1* RNAi. Animals were allowed to develop until late larval stage L4 (GFP::RAB-10 and GFP::RAB-11.1) or day 3 of adulthood (ACT-5::GFP). Worms were then rinsed off plates with M9 buffer, centrifuged (1,000 x g) for 30 s, and washed twice with M9 buffer before being transferred to 1.5 mL Eppendorf tubes and then rapidly flash frozen in liquid nitrogen. Worm extracts were generated by glass bead disruption in non-denaturing lysis buffer [50 mM Hepes pH 7.4, 150 mM NaCl, 1mM EDTA, 1% Triton X-100, EDTA-free mini-protease inhibitor cocktail (Roche)]. Crude lysates were subject to centrifugation at 8,000 x g at 4°C for 5 min. For ACT-5::GFP sample preparation, lysates were subject to ultracentrifugation at 150,000 x g at 4°C for 30 min to isolate the soluble (supernatant) and insoluble (pellet) protein fractions. For immunoprecipitations, samples were transferred to 1.5 mL Eppendorf tubes and supplemented with 5 μL (2 μg) of mouse anti-GFP monoclonal antibody (Roche 11814460001) and incubated for 1 h. Next, 100 μL of Protein G magnetic beads (Bio-Rad 1614023) were added and the lysate-antibody mixture was rotated 360 degrees for 1 h at 4°C. Following incubation, the G-protein beads were magnetically precipitated and gently washed three times with 1 mL of non-denaturing lysis buffer before being resuspended in 30 mM TEAB (Triethylammonium bicarbonate) buffer containing 4% SDS.

To prepare samples for LC-MS/MS analysis, disulfide bonds were reduced with 20 mM DTT, followed by alkylation with 40 mM IAA (Iodoacetamide) and acidification by phosphoric acid. Samples were diluted in binding buffer (1 to 9 volume of TEAB to methanol) and loaded on S-Trap^™^ micro columns (Protifi). Proteins were digested with a ratio of 1/25 trypsin (Pierce) overnight at 37°C and eluted peptides were dried out in a speedvac and reconstituted in 2% ACN (Acetonitrile) and 0.1% TFA (Trifluoroacetic acid). Peptides were separated using an UltiMate 3000 RSLC nano LC system (Thermo Fisher Scientific) equipped with an EASY-Spray^™^ 150 mm column with 75 μM inner diameter (Thermo Fisher Scientific ES800A) heated to 55°C. Gradient elution was performed from 2% acetonitrile to 30% acetonitrile in 0.1% formic acid over 90 min. Q-Exactive HF mass spectrometer was set to acquire data in a data-dependent top 20 method with the full-MS scans acquired at 120 K resolution and MS/MS scans acquired at 15 K resolution (at *m/z* 200). Normalized collision energy was 28 and the minimum AGC trigger was 2e^2^ ions (intensity threshold 1e^3^). The peptide match algorithm was set to preferred and charge exclusion was applied to exclude unassigned peptides. Charge and dynamic exclusion were applied with a duration of 20 s.

Proteomics searches were performed using the *C. elegans* reference database from UniProt using Proteome Discoverer 2.4. Peptides with a minimum of six amino acid residues with trypsin/P specificity were identified. Protein N-terminal acetylation and carbamidomethyl of cysteine were used as fixed modifications and methionine oxidation was used as a variable modification. Spectral indexes for interacting proteins were analyzed as described below.

#### Rab GTPase protein interaction analysis

For analyses of GFP::RAB-10 and GFP::RAB-11.1 co-immunoprecipitation analysis by LC-MS/MS, RAB-10 and RAB-11.1 levels were normalized by spectral index for control and *hsf-1* RNAi conditions, and interacting protein abundance was adjusted accordingly. The mean spectral index of the two biological replicates was used to calculate relative binding (*hsf-1* RNAi/control RNAi) for each interacting protein. Proteins were considered enriched if peptide spectral match (PSM) ≥ 2. DAVID Bioinformatics Resources 6.8 (Huang et al., 2009a; 2009b) was used for KEGG pathway analysis of proteins enriched in GFP::RAB-11.1 co-immunoprecipitations. Relative binding to endocytosis-related (cel04144) proteins was compared for control and *hsf-1* RNAi conditions. Statistical analysis was performed using Prism 9. See Table S6 for peptide enrichment in GFP::RAB-10 and GFP::RAB-11.1 co-immunoprecipitations.

#### Solubility ratio and destabilization indexing

For ACT-5::GFP co-immunoprecipitation analysis, a solubility ratio for each ACT-5 interacting protein was calculated for both control and *hsf-1* RNAi conditions by ratioing the spectral index in the soluble fraction over the total spectral index from both fractions. Destabilization index values were determined for each ACT-5::GFP interacting protein by subtracting the solubility ratio in *hsf-1* RNAi conditions from that of control RNAi conditions. Destabilization index values ranged from −1 to 1, with 1 representing a complete shift from the soluble fraction in control RNAi conditions to the insoluble fraction in *hsf-1* RNAi conditions. DAVID Bioinformatics Resources 6.8 (Huang et al., 2009a; 2009b) was used for GO term analysis of the ACT-5 interacting proteins with the highest destabilization indexes (top 10%). See Table S4 for list of proteins with the top 10% highest scores. See Table S7 for solubility ratios and destabilization index analysis of all ACT-5::GFP interacting proteins.

#### Sample preparation for western blotting

All western blots were performed on day 3 adult worms cultivated on nematode growth media (NGM) agarose plates supplemented with HT115 *E. coli* at 20°C. Animals were rinsed off plates with liquid M9 buffer, centrifuged at 1,000 x g for 30 s in a room temperature clinical centrifuge, and washed twice with M9 before being transferred to 1.5 mL Eppendorf tubes and rapidly flash frozen in liquid nitrogen. Frozen worm pellets were thawed on ice and worm extracts were generated by glass/zirconia bead disruption in non-denaturing lysis buffer [50 mM Hepes pH 7.4, 150 mM NaCl, 1mM EDTA, 1% Triton, EDTA-free mini-protease inhibitor cocktail (Roche)]. All worm extracts were subject to centrifugation at 8,000 x g at 4°C for 5 min prior to protein determination with a BCA protein quantification kit (Thermo Scientific). For ACT-5::GFP solubility analysis, samples were subject to ultracentrifugation at 150,000 x g at 4°C for 30 min to isolate the soluble (supernatant) and insoluble (pellet) protein fractions. For limited proteolysis analysis of ACT-5::GFP, equal volumes (40 μL) of the pre-cleared lysates were aliquoted into six 1.5 mL Eppendorf tubes and treated with 1.5 μL of 1x non-denaturing lysis buffer containing various concentrations of trypsin [0.0, 0.1, 0.5, 1.0, and 2.5 mg/mL] for 20 min on ice. Samples were then supplemented with 2x Laemmli sample buffer and boiled at 90°C for 10 min.

#### Filter trap analysis and western blotting

Filter trap and Western blot analyses were performed from the same lysates, split equally into separate 1.5 mL Eppendorf tubes. For filter trap analysis, 5% SDS was added to samples for a final concentration of 0.5% SDS. The Bio-Dot SF slot blot apparatus (Bio-Rad 1706542) was assembled with a 0.45-μm pore size cellulose acetate membrane (Sterlitech CA0453001) atop pre-soaked Whatman paper filters, vacuumed to tighten, and equilibrated for 1 h with 100 μL of equilibration buffer [1x lysis buffer with 0.5% SDS] per well. Samples were vortexed, loaded onto equilibrated wells, and allowed to flow through by gravity. Once dry, wells were washed with 100 μL of 0.1% SDS lysis buffer and allowed to dry prior to slot blot apparatus disassembly. Membranes were stained as described below. For Western blot analysis, lysates were supplemented with 2x Laemmli sample buffer and boiled at 90°C for 10 min. Samples were resolved by SDS-PAGE, transferred to 0.22-μm nitrocellulose membranes, and subject to Western blot analysis (Egge et al., 2019). Electrophoresis with 10% SDS-PAGE gels was performed at a constant 80 V until the 37 kDa protein standard of the prestained protein ladder (EZ-RUN, Thermo-Fisher) reached the bottom of the gels.

All antibodies were prepared in 5% BSA/PBST. Rabbit anti-α-Tubulin (Abcam ab4074) and secondary goat anti-rabbit (LiCor 926-32211) antibodies were used at 1:10,000. Mouse anti-GFP (Roche 1814460001 at 0.4 mg/mL) and secondary goat anti-mouse (LiCor 926-68070) antibodies were used at 1:5,000. The rabbit monoclonal anti-ERM-1 (Developmental Studies Hybridoma Bank ERM1) antibody was used at a 1:1000 dilution. Western and slot blots were imaged and quantified using Image Studio software (LI-COR Biosciences, Lincoln, NE). For limited proteolysis analysis, ACT-5::GFP or ERM-1 signal intensity in trypsin-digested bands were compared to that of untreated control for each RNAi condition. Samples were run in triplicate and ratioed band intensities were averaged for each biological replicate. Prism 9 was used for statistical analysis.

### QUANTIFICATION AND STATISTICAL ANALYSIS

Statistical details of all experiments, including exact n values (number of biological and technical replicates), the use of precision measures (e.g. SEM), type of statistical test performed and significance values, are described in the figure legends. Statistical analyses, including student’s t-test, log-rank (Mantel-Cox), and ANOVA with post-hoc multiple comparisons analysis, were conducted using Prism (version 9.2.0, GraphPad) and CLC Genomics Workbench (version 9.5). *p*-values < 0.05 were considered significant.

## Supplementary Material

1

2

3

## Figures and Tables

**Table:** KEY RESOURCES TABLE

REAGENT or RESOURCE	SOURCE	IDENTIFIER
Antibodies		
Mouse monoclonal anti-GFP	Sigma-Aldrich	Cat#11814460001 (Roche); RRID: AB_390913
Rabbit polyclonal anti-α tubulin	Abcam	Cat#ab4074; RRID: AB_2288001
Rabbit monoclonal anti-ERM-1	Developmental Studies Hybridoma Bank	Cat#ERM1; RRID: AB_10584795
Goat anti-mouse	LiCor	Cat#926-68070; RRID: AB_10956588
Goat anti-rabbit	LiCor	Cat#926-32211; RRID: AB_621843
Bacterial and virus strains		
HT115 (DE3) *Escherichia coli*	(Rual et al., 2004)	CGC Cat#HT115 (DE3), RRID: WB-STRAIN: HT115(DE3)
OP50 *Escherichia coli*	(Brenner, 1974)	CGC Cat#OP50, RRID: WB-STRAIN: OP50
Chemicals, peptides, and recombinant proteins		
Sodium cacodylate buffer, 0.2M, pH7.4	Electron Microscopy Sciences	Cat#11650
Osmium tetroxide	Electron Microscopy Sciences	Cat#19100
Uranyl acetate solution, 2%	Electron Microscopy Sciences	Cat#224004
Spurr’s resin	Electron Microscopy Sciences	Cat#14300
Propylene oxide	Electron Microscopy Sciences	Cat#20401
Lead citrate	Electron Microscopy Sciences	Cat#17800
Levamisole, 99%	Acros Organics	Cat#187870100
Proteinase K	VWR	Cat#97062-670
Complete, EDTA-free mini protease inhibitor cocktail	Sigma-Aldrich	Cat#11836170001
EZ-Run prestained rec protein ladder	Thermo Fisher Scientific	Cat#BP36031
Alfa Aesar oil red O	Thermo Fisher Scientific	Cat#AAA1298914
AUTODOT visualization dye	Abcepta	Cat#SM1000a
TRIzol	Thermo Fisher Scientific	Cat#15-596-026
SureBeads protein G magnetic beads	Bio-Rad	Cat#161-4023
COPAS GP sheath reagent	Union Biometrica	Cat#300-5070-100
Albumin from Bovine Serum (BSA), Tetramethylrhodamine (TRITC) conjugate	Thermo Fisher Scientific	Cat#A23106
Invitrogen FM4-64 Dye	Thermo Fisher Scientific	Cat#T3166
Critical commercial assays		
QuantiTect Reverse Transcription kit	Qiagen	Cat#205313
iTaq Universal SYBR Green Supermix kit	Bio-Rad	Cat#1725124
Deposited data		
Raw RNA sequencing data (heat shock and *hsf-1* RNAi)	(Brunquell et al., 2016)	NCBI BioProject Repository: PRJNA311958
Raw microarray data (*hsf-1* OE)	(Baird et al., 2014)	N/A
Raw RNA sequencing data (WT vs *nhr-49(nr2041)*)	This paper	GeoDatasets: GSE199971
Chromatin immunoprecipitation sequencing for HSF-1	(Li et al., 2016)	GeoDatasets: GSE81521
Experimental models: Organisms/strains		
*S. cerevisiae*: BY4742	Horizon a PerkinElmer company	Cat#YSC1049
*S. cerevisiae*: BY4742 *hsf1*-DAmP strain	Horizon a PerkinElmer company	Cat#YSC5090
*C. elegans*: Strain N2 (ancestral) as wild-type (WT)	Caenorhabditis Genetics Center	CGC Cat#N2 (ancestral), RRID: WB-STRAIN: N2_(ancestral)
*C. elegans*: Strain CF512: *rrf-3(b26) II; fem-1(hc17) IV*	Caenorhabditis Genetics Center	CGC Cat#CF512, RRID: WB-STRAIN: CF512
*C. elegans*: Strain AGD710: *uthIs235[sur5-p::hsf-1 + myo-2p::tdTomato*]	Caenorhabditis Genetics Center	CGC Cat#AGD710, RRID: WB-STRAIN: AGD710
*C. elegans*: Strain STE68: *nhr-49(nr2041) I*	Caenorhabditis Genetics Center	CGC Cat#STE68, RRID: WB-STRAIN: STE68
*C. elegans*: Strain LIU1: *ldrIs1[dhs-3p::dhs-3::gfp + unc-76(+)]*	Caenorhabditis Genetics Center	CGC Cat#WBM170, RRID: WB-STRAIN: WBM170
*C. elegans*: Strain WBM170: *wbmEx57[acs-2p::gfp + rol-6(su1006)]*	Caenorhabditis Genetics Center	CGC Cat#WBM170, RRID: WB-STRAIN: WBM170
*C. elegans*: Strain CL2070: *dvln70[hsp-16.2p::gfp + rol-6(su1006)]*	Caenorhabditis Genetics Center	CGC Cat#CL2070, RRID: WB-STRAIN: CL2070
*C. elegans*: Strain RT311: *pwls69[vha-6p::gfp::rab-11.1 + unc-119(+)]*	Caenorhabditis Genetics Center	CGC Cat#RT311, RRID: WB-STRAIN: RT311
*C. elegans*: Strain RT327: *pwIs72[vha-6p::gfp::rab-5 + Cbr-unc-119(+)] II*	Caenorhabditis Genetics Center	CGC Cat#RT327, RRID: WB-STRAIN: RT327
*C. elegans*: Strain RT476: *pwIs170 [vha6p::gfp::rab-7 + Cbr-unc-119(+)])*	Caenorhabditis Genetics Center	CGC Cat#RT476, RRID: WB-STRAIN: RT476
*C. elegans*: Strain RT525: *pwIs206 [vha6p::gfp::rab-10 + Cbr-unc-119(+)]*	Caenorhabditis Genetics Center	CGC Cat#RT525, RRID: WB-STRAIN: RT525
*C. elegans*: Strain OG472: *drSi2[vha-6p::3xflag::pgp-3/CFTR(wt)::mCherry] II*	Caenorhabditis Genetics Center	CGC Cat#OG472, RRID: WB-STRAIN: OG472
*C. elegans*: Strain AGP33a: *glmEx8(nhr-49p::nhr-49::gfp + myo3p::mCherry); nhr-49(nr2041) I*	Laboratory of Arjumand Ghazi (Ratnappan et al., 2014)	N/A
*C. elegans*: Strain MZE1: *unc-119(ed3) III; cbgIs91[pept-1p::pept-1::DsRed + unc-119(+)]; rrf-3(pk1426) II; pwls69[vha-6p::gfp::rab-11 + unc-119(+)]*	Laboratory of Marino Zerial (Winter et al., 2012)	N/A
*C. elegans*: Strain ERT137: *jyIs17[act-5p::mCherry::act-5 + ttx-3p::rfp] IV; dkIs8 [chc-1p::chc-1::gfp]*	Laboratory of Emily Troemel (Szumowski et al., 2016)	N/A
*C. elegans*: Strain PMD19: *jyIs13x3 [gfp::act-5] II; fer-15(b26) II; fem-1(hc17ts) IV*	(Egge et al., 2019)	N/A
*C. elegans*: Strain PHX2999: *rab-11.2(syb2999) I*	(Watterson et al., 2022)	N/A
*C. elegans*: Strain PMD90: *fem-1(hc17ts) IV; pwls209[vha-6p::gfp::rab-11.1 + Cbr-unc-119(+)]*	(Watterson et al., 2022)	N/A
*C. elegans*: Strain PMD91: *fem-1(hc17ts) IV; pwls69[vha-6p::gfp::rab-10 + Cbr-unc-119(+)]*	(Watterson et al., 2022)	N/A
*C. elegans*: Strain PMD118: *utsEx14[rab-11.2p::yfp]; nhr-49(nr2041) I*	(Watterson et al., 2022)	N/A
*C. elegans*: Strain PMD124: *utsIs3[rab-11.2p::yfp]*	(Watterson et al., 2022)	N/A
*C. elegans*: Strain PMD150: *utsIs4 [glmEx8(nhr-49p::nhr-49::GFP + myo3p::mCherry]; nhr-49(nr2041) I*	(Watterson et al., 2022)	N/A
*C. elegans*: Strain PMD169: *cbgIs91[pept-1p::pept-1::DsRed + unc-119(+)]; rrf-3(pk1426) II; utsIs6[rab-11.2(syb2999)]*	(Watterson et al., 2022)	N/A
*C. elegans*: Strain PMD170: *ldrIs1[dhs-3p::dhs-3::gfp + unc-76(+)];rab-11.2(syb2999) I*	(Watterson et al., 2022)	N/A
*C. elegans*: Strain PMD17: *pwls69 [vha-6p::gfp::rab-11 + unc-119(+)] ;jyIs17[act-5p::mCherry::act-5 + ttx-3p::rfp] IV*	This paper	N/A
*C. elegans*: Strain PMD142: *ldrIs1[dhs-3p::dhs-3::gfp + unc-76(+)]; nhr-49(nr2041) I*	This paper	N/A
*C. elegans*: Strain PMD157: *ldrIs1[dhs-3p::dhs-3::gfp + unc-76(+)]; uthIs235[sur5-p::hsf-1 + myo-2p::tdTomato]*	This paper	N/A
*C. elegans*: Strain PMD121: *N2; wild-type nhr-49*	This paper	N/A
*C. elegans*: Strain PMD122: *N2; nhr-49(nr2041) I*	This paper	N/A
*C. elegans*: Strain PMD168: *wbmEx57[acs-2p::gfp + rol-6(su1006)]; uthIs235[sur5-p::hsf-1 + myo-2p::tdTomato]*	This paper	N/A
Oligonucleotides		
qPCR primer: *tba-1*: Forward: TCCACTGATCTCTGCTGACAA	(Egge et al., 2021)	N/A
qPCR primer: *tba-1*: Reverse: TGGATCGCACTTCACCATT	(Egge et al., 2021)	N/A
qPCR primer: *Y45F10D.4*: Forward: TCTTCCCTGGCAACCGAATG	(Egge et al., 2021)	N/A
qPCR primer: *Y45F10D.4*: Reverse: CTTGGGCGAGCATTGAACAG	(Egge et al., 2021)	N/A
qPCR primer: *rab-11.1*: Forward: GAGTCGAGTTTGCCACGAGA	This paper	N/A
qPCR primer: *rab-11.1*: Reverse: CACGGTAACGTTCCTGTCCA	This paper	N/A
qPCR primer: *rab-11.2*: Forward: TTGGGATACGGCTGGAATGG	This paper	N/A
qPCR primer: *rab-11.2*: Reverse: TCACGAAGCACCTTCAACCA	This paper	N/A
*nhr-49(nr2041)* genotyping primer: Forward: CTTTCTTTCCTTTTCCTGTCCGT	(Watterson et al., 2022)	N/A
*nhr-49(nr2041)* genotyping primer: Reverse: ATGAGATGTTGTGGTGCATAGT	(Watterson et al., 2022)	N/A
*rab-11.2(syb2999)* genotyping primer: Forward: CTCACCTTCCCTTTTTCTGG	(Watterson et al., 2022)	N/A
*rab-11.2(syb2999)* genotyping primer: Reverse: CGTCATTTCATAAACAATTTGGCCCC	(Watterson et al., 2022)	N/A
RNAi targeting sequence: empty vector (control)	L4440 plasmid	N/A
RNAi targeting sequence: *hsf-1*: TCTAGAAAATTCCGGGAAAAACT	Ahringer library	N/A
RNAi targeting sequence: *nhr-49*: N/A	Vidal library	N/A
RNAi targeting sequence: *act-5*: CCTGCTTGGAGATCCACATT	Ahringer library	N/A
RNAi targeting sequence: *rfip-2*: GTGGCCTCTACATGAAGAACAAG	Ahringer library	N/A
RNAi targeting sequence: *myo-1*: ATTGGCGGGTAAGTCTGTTG	Ahringer library	N/A
RNAi targeting sequence: *myo-3*: TTCGAGAGAGCGGTTCAAAT	Ahringer library	N/A
RNAi targeting sequence: *myo-5*: AGCTCCGTCAAGACTTGGAA	Ahringer library	N/A
RNAi targeting sequence: *nmy-1*: TCTAAGAGCCACTTTGCCGT	Ahringer library	N/A
RNAi targeting sequence: *nmy-2*: TCCGAGAAGTGAAGCGATTT	Ahringer library	N/A
Software and algorithms		
ZEN Software (ver. 2.3)	Carl Zeiss Microscopy, LLC	https://www.zeiss.com/microscopy/us/products/microscope-software/zen.html
Leica Application Suite X	Leica	https://www.leica-microsystems.com/products/microscope-software/p/leica-las-x-ls/
Prism (ver. 9.2.0 for Windows)	GraphPad	https://www.graphpad.com
Fiji Software	ImageJ	https://imagej.net/software/fiji/
CLC Genomics Workbench (ver. 9.5)	Qiagen Bioinformatics	https://www.qiagenbioinformatics.com/products/clc-genomics-workbench/
Proteome Discoverer Software (ver. 2.4)	Thermo Fisher Scientific	https://www.thermofisher.com/order/catalog/product/OPTON-30957
Image Studio Software (ver. 5.0)	LI-COR Biosciences	https://www.licor.com/bio/image-studio/
FlowPilot Software (ver. 2.6.1)	Union Biometrica	https://www.unionbio.com/copas/
DAVID Bioinformatics Resources (ver. 6.8)	(Huang et al., 2009a; 2009b)	https://david.ncifcrf.gov/
Gene List Venn Diagram Generator	Source Forge	http://genevenn.sourceforge.net/
Analyst Software (ver. 1.7)	SCIEX	https://sciex.com/products/software/analyst-software

## Highlights

- Lipid deposition is modulated by HSF-1 levels in *C. elegans*
- Lifespan extension by HSF-1 requires NHR-49
- Reduced *hsf-1* expression impairs membrane trafficking through actin destabilization
- Loss of *hsf-1* activates the intracellular lipid surveillance response
