# Supplementary material for: Loss of heat shock factor initiates intracellular lipid surveillance by actin destabilization
Source: Cell Rep. Author manuscript; Available in PMC 2022 Nov 8. (PMC9642076; doi:10.1016/j.celrep.2022.111493)
Supplement: 1 [file NIHMS1843692-supplement-1.pdf]

Cell Reports, Volume *41*

Supplemental information

Loss of heat shock factor initiates intracellular lipid surveillance by actin destabilization

Abigail Watterson, Sonja L.B. Arneaud, Naureen Wajahat, Jordan M. Wall, Lexus Tatge, Shaghayegh T. Beheshti, Melina Mihelakis, Nicholas Y. Cheatwood, Jacob McClendon, Atossa Ghorashi, Ishmael Dehghan, Chase D. Corley, Jeffrey G. McDonald, and Peter M. Douglas

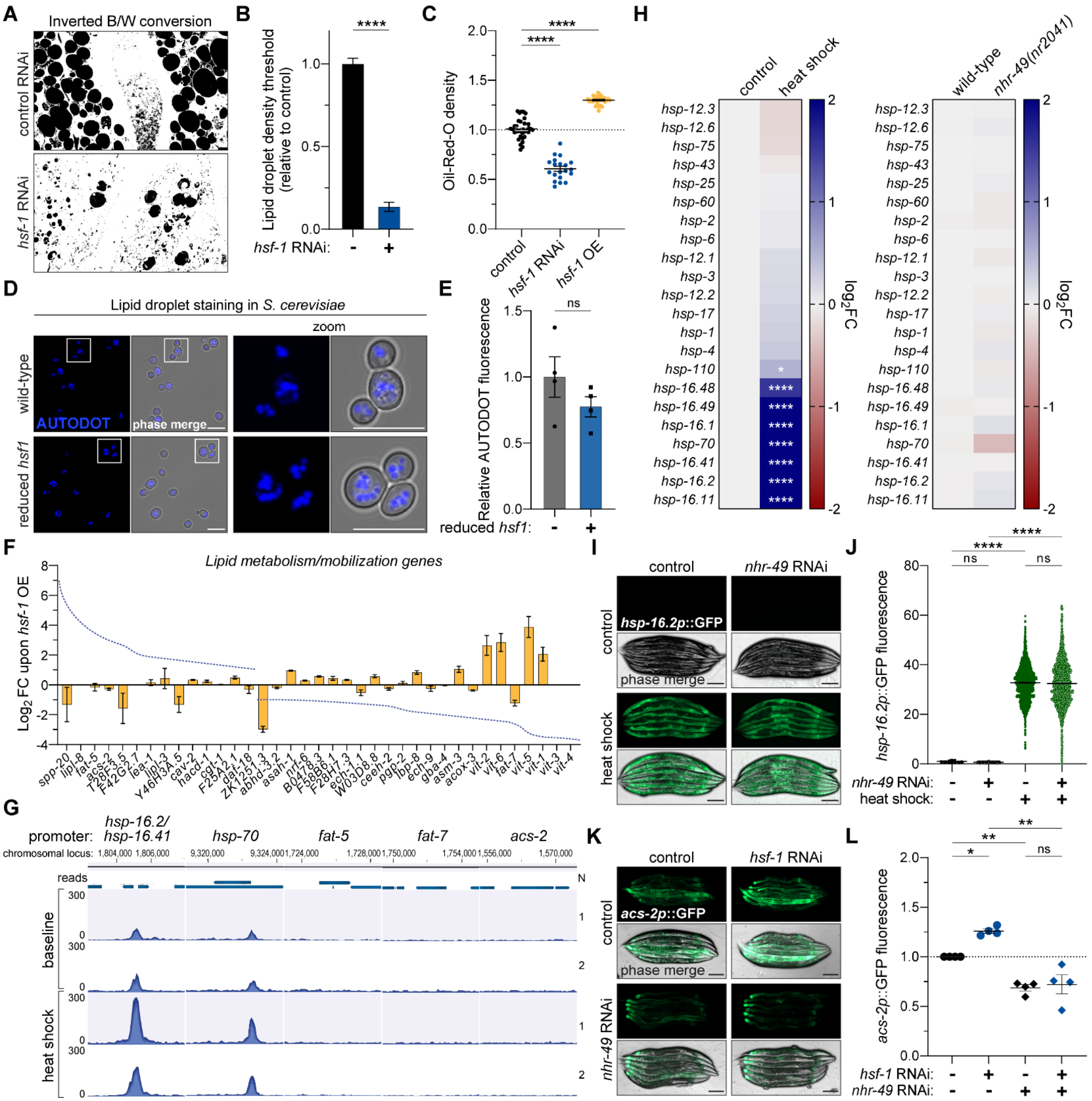

Supplemental Figure S1. HSF-1 modulates lipid deposition and NHR-49 target gene expression in *C. elegans*. Related to Figure 1. (**A,B**) Quantification of intestinal lipid droplet density from TEM micrographs of adult *C. elegans* intestinal cross sections subject to black/white conversion. Animals were treated with empty vector control or *hsf-1* RNAi. **A**, Example of black/white-converted micrographs used for density threshold analysis. **B**, Relative lipid droplet density threshold, measured from histogram quantification of black saturation. Mean ± SEM for 10 micrographs per condition. ****$p < 0.0001$ by two-tailed unpaired t-test. (**C**) Relative Oil-Red-O staining density measured from micrographs of day 5 adult *C. elegans*. Mean ± SEM for 34 (control), 20 (*hsf-1* RNAi), and 31 (*hsf-1* OE) animals across 3 replicate experiments. ****$p < 0.0001$ by one-way ANOVA with Tukey's multiple comparisons. (**D,E**) AUTODOT lipid droplet staining in *S. cerevisiae* with or without reduced *hsf1* expression. **D**, Representative micrographs. Scale = 10 µm. **E**, Relative fluorescence by fluorimetry.

Mean ± SEM for 3 replicate experiments. ns = not significant by two-tailed unpaired t-test. (**F**) Relative expression of genes involved in lipid/fatty acid metabolism and mobilization upon *hsf-1* OE. Values represent $\log_2$FC transcript abundance, detected by RNA-seq, relative to wild-type control. Blue line represents $\log_2$FC of the same genes upon *hsf-1* RNAi (see Figure 1E). See also Table S1 for statistics. (**G**) HSF-1 binding to promoter elements within the 5' untranslated regions (UTR) of select genes involved in thermotolerance and lipid metabolism at baseline (20°C), top, and heat shock (33°C), bottom, conditions, detected by chromatin immunoprecipitation sequencing (ChIP-seq) analysis of OG497 worms (Li *et al.*, 2016). Shown are peaks from 2 biological replicates. (**H**) Heatmaps display relative fold change of heat shock protein transcript abundance upon heat shock, left, or in *nhr-49(nr2041)* mutants, right, compared to the respective controls. Colorimetric scale corresponds to relative $\log_2$FC. Means from 2 (left) or 3 (right) biological replicates. **p* = 0.0393, *****p* < 0.0001 by two-way ANOVA with Sidak's multiple comparisons. (**I,J**) Fluorescence of the *hsp-16.2p*::GFP transcriptional reporter in *C. elegans* on control or *nhr-49* RNAi following treatment with or without heat shock for 40 min at 33°C and 4-hour recovery at 20°C. **I**, Fluorescence micrographs. Scale = 200 µm. **J**, Relative fluorescence by flow cytometry. Mean ± SEM for, from left to right, 2,802, 2,907, 3,019 and 3,420 animals across 3 replicate experiments. ns = not significant, *****p* < 0.0001 by one-way ANOVA with Tukey's multiple comparisons. (**K,L**) Transgenic adult *C. elegans* harboring the *acs-2p*::GFP transcriptional reporter treated with control, *nhr-49*, and/or *hsf-1* RNAi. **K**, Fluorescence micrographs. Scale = 200 µm. **L**, Relative *acs-2p*::GFP fluorescence by flow cytometry. Mean ± SEM for 4 replicate experiments. ns = not significant, **p* = 0.0106, ***p* = 0.0029 and 0.0065 by two-way ANOVA with Tukey's multiple comparisons.

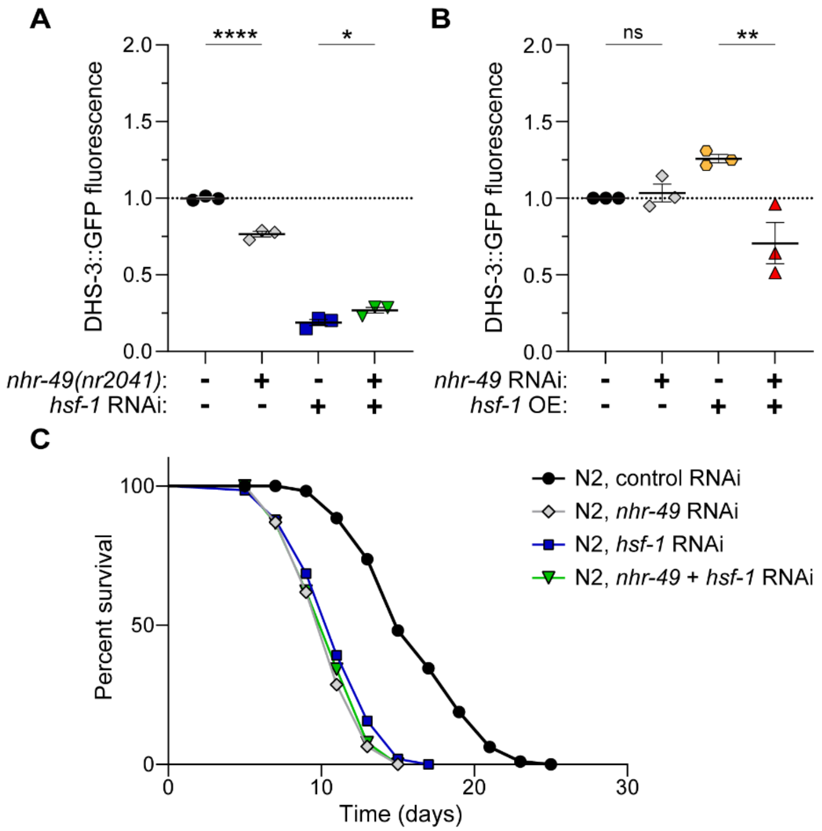

Supplemental Figure S2. HSF-1 impacts lipid deposition and age through NHR-49. Related to Figure 2. (**A,B**) Relative DHS-3::GFP fluorescence by flow cytometry in day 5 adult wild-type and *nhr-49(nr2041)* mutant *C. elegans* treated with control or *hsf-1* RNAi, **A**, and wild-type or *hsf-1* OE *C. elegans* treated with control or *nhr-49* RNAi, **B**. Mean ± SEM for 3 replicate experiments. *$p = 0.0404$, ****$p < 0.0001$ (**A**), ns = not significant, **$p = 0.0046$ (**B**) by two-way ANOVA with Sidak's multiple comparisons. (**C**) Lifespan curves for N2 (wild-type) worms cultured on control, *nhr-49*, and/or *hsf-1* RNAi. Shown are representative lifespans of 3 replicates with approximately 100 animals per condition (see Table S3). $p < 0.0001$ by log-rank test.

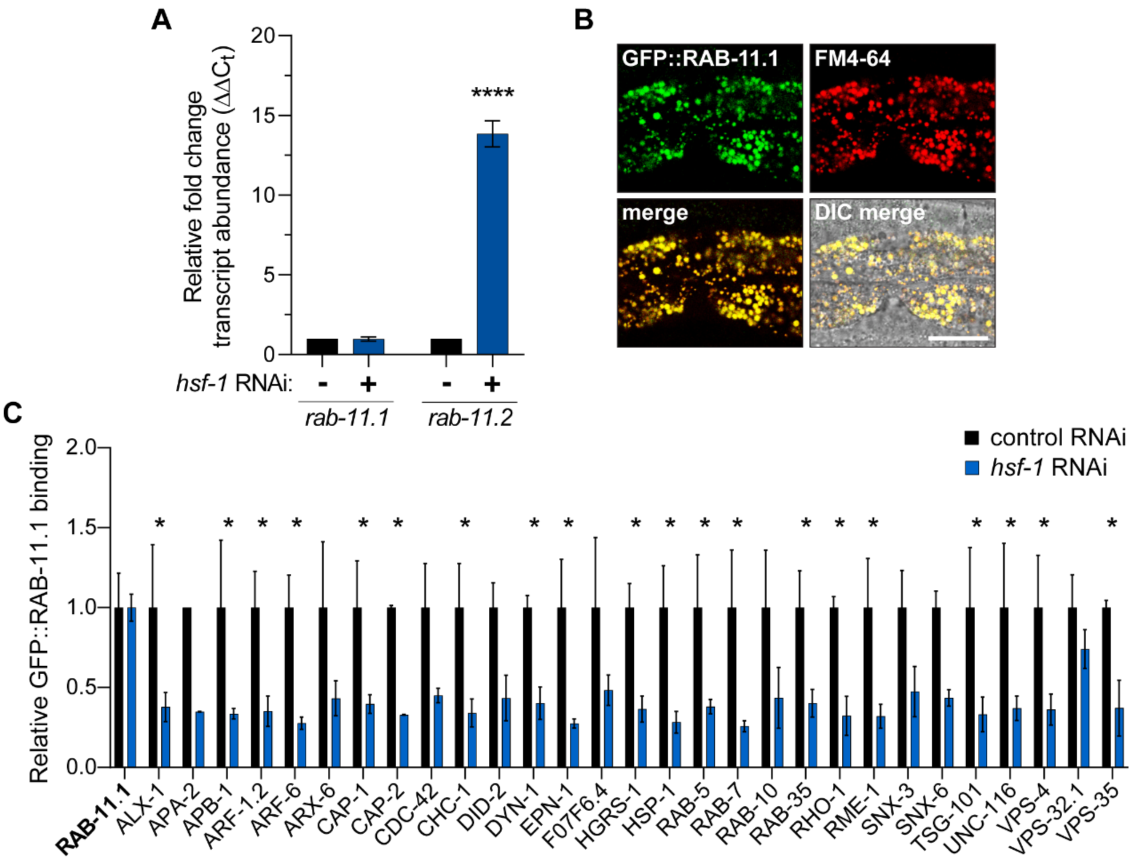

Supplemental Figure S3. Loss of *hsf-1* reduces RAB-11.1 binding to endocytic vesicle components. Related to Figure 3. (**A**) Relative transcript abundance compared to control RNAi, based on normalized cycle threshold (C_t) values detected by qPCR. Mean ± SEM for 3 biological replicates. ****$p < 0.0001$ by two-way ANOVA with Tukey's multiple comparisons. (**B**) Localization of GFP::RAB-11.1 and lipophilic endocytic vesicle marker, FM4-64, in *C. elegans* intestinal epithelia. Scale = 25 µm. (**C**) Relative enrichment of endocytosis-related proteins co-immunoprecipitated with GFP::RAB-11.1 and detected by LC-MS/MS. Peptide abundance was standardized according to GFP::RAB-11.1 levels. Mean ± SEM for 2 biological replicates. *$p < 0.05$ by multiple two-tailed unpaired t-tests.

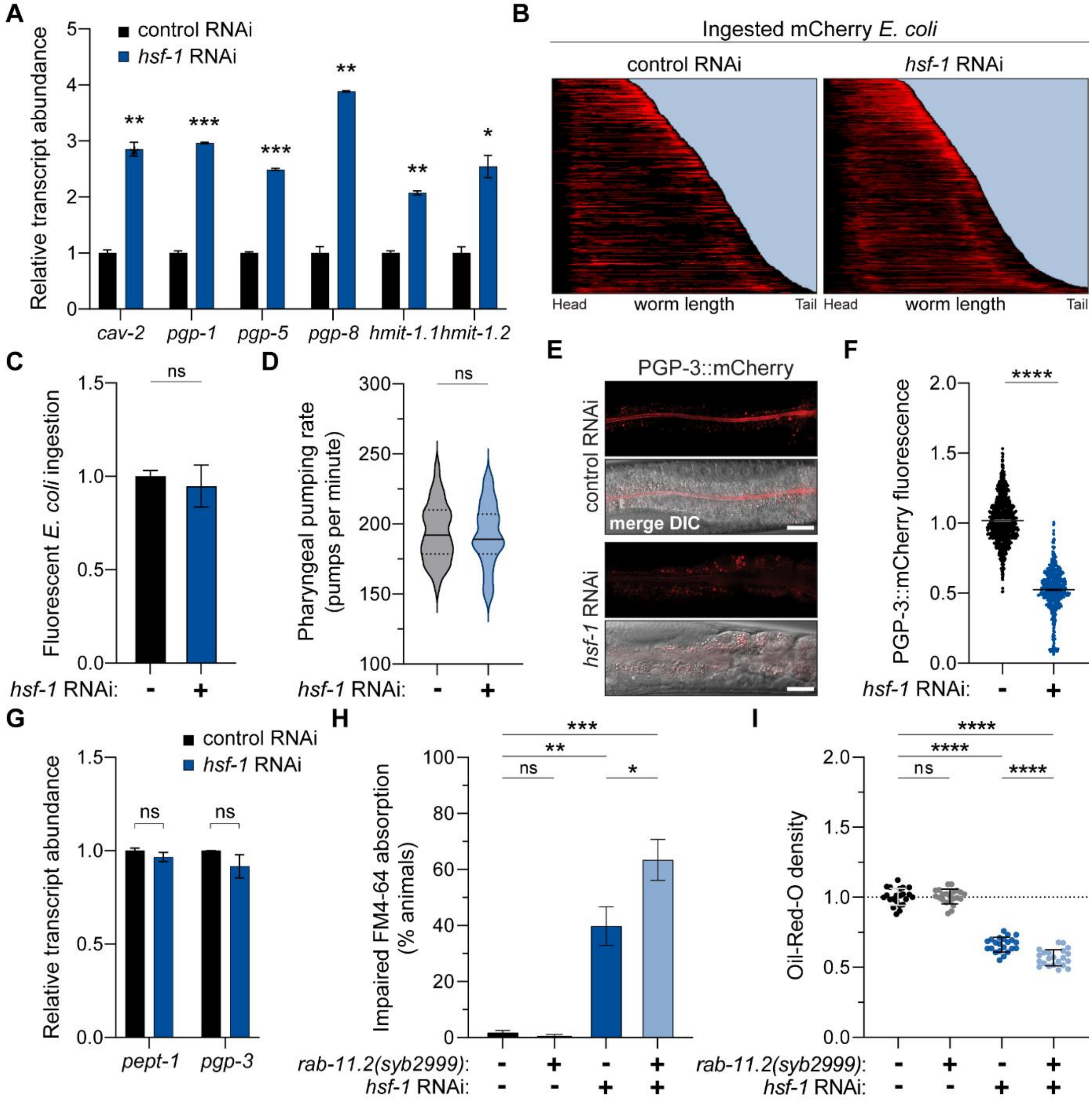

Supplemental Figure S4. Adaptive response to impaired membrane recycling and absorption by *hsf-1* RNAi. Related to Figure 4. (**A-I**) Comparative analyses of adult *C. elegans* treated with control or *hsf-1* RNAi. (**A**) Relative fold change in transcript abundance of apical intestinal transporters upon *hsf-1* RNAi. Mean ± SEM for 2 biological replicates. $p = 0.0053$ (*cav-2*), 0.0004 (*pgp-1* and *pgp-5*), 0.0017 (*pgp-8*), 0.0025 (*hmit-1.1*) and 0.0211 (*hmit-1.2*) by multiple two-tailed unpaired t-tests. (**B,C**) Ingestion of fluorescent *E. coli* in day 1 adults. *C. elegans* were analyzed by flow cytometry immediately following media supplementation with mCherry-expressing *E. coli*. **B**, Fluorescence profiles of individual animals, aligned from top to bottom according to length (time-of-flight). Shown are profiles of 1,484, left, and 1,582, right, animals. **C**, Relative fluorescence. Mean ± SEM for 3 replicate experiments. ns = not significant by two-tailed unpaired t-test. (**D**) Pharyngeal pumping rate (pumps per minute). Mean ± SEM for 30 animals across 3 replicate experiments. $p = 0.8507$ by two-tailed unpaired t-test, ns = not significant. (**E,F**) Expression of apical transporter, PGP-3::mCherry. **E**, Representative micrographs. Scale = 25 µm. **F**, Relative PGP-3::mCherry fluorescence by flow cytometry. Mean ± SEM for 996 (control RNAi) and 701 (*hsf-1* RNAi) animals across 3 replicate experiments. **$p = 0.0029$ by two-tailed unpaired t-test. (**G**) Relative fold change in transcript abundance upon *hsf-1* RNAi. Mean ± SEM for 2 biological replicates. $p = 0.3602$ (*pept-1*) and 0.3067 (*pgp-3*) by multiple two-tailed unpaired t-tests. (**H**) Relative ratio of animals which absorbed less than 50% of ingested FM4-64 into the intestinal epithelia following its dietary

supplementation in adult wild-type and *rab-11.2(syb2999)* mutant *C. elegans*. Mean ± SEM for 3 replicate experiments (75 animals per condition). *p = 0.0429, **p = 0.0031, ***p = 0.0001 by two-way ANOVA with Tukey's multiple comparisons. (**I**) Relative Oil-Red-O staining density measured from day 5 adult wild-type and *rab-11.2(syb2999) C. elegans*. Mean ± SEM for 22 animals per condition across 3 replicate experiments. ****p < 0.0001 by one-way ANOVA with Tukey's multiple comparisons.

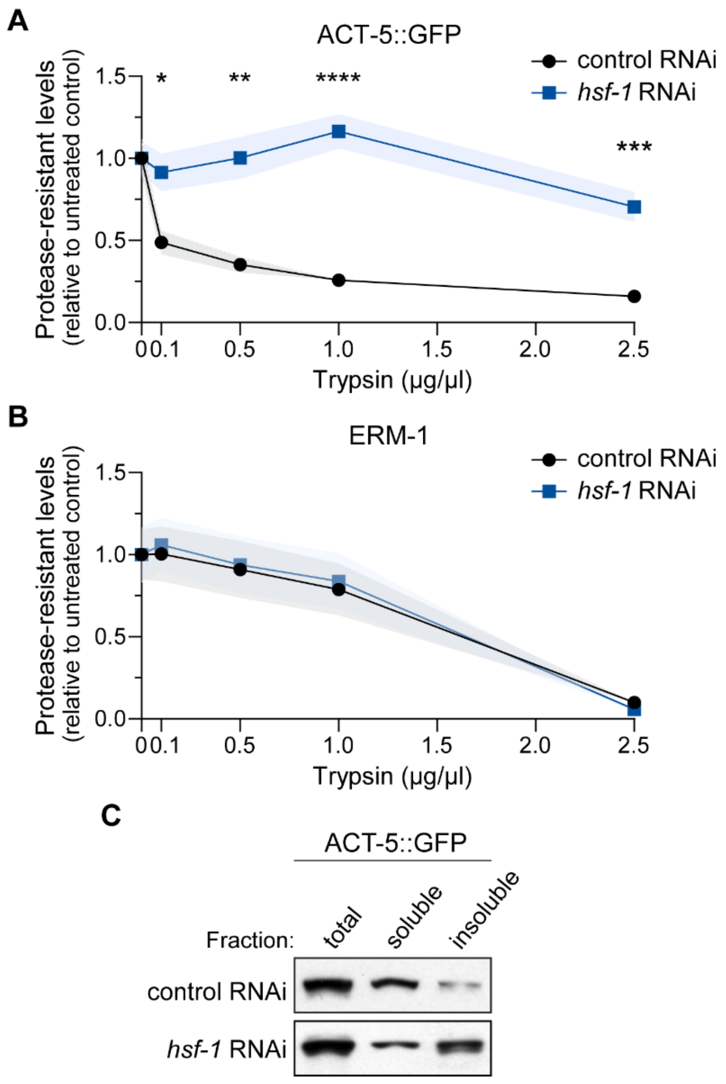

Supplemental Figure S5. Loss of *hsf-1* drives enteric actin insolubility. Related to Figure 5. (**A,B**) Protease resistance of ACT-5::GFP, **A**, and ERM-1, **B**, in control versus *hsf-1* RNAi conditions. Lysates from day 1 adult *C. elegans* were treated with increasing concentrations of trypsin and resolved by western blot (see Figure 5B). Shown is mean ± SEM (shaded regions) of signal intensity in each treatment condition relative to untreated control from 3 replicate experiments. *p = 0.0142, **p = 0.0015, ***p = 0.0002, ****p < 0.0001 by two-way ANOVA with Sidak's multiple comparisons. (**C**) Western blot analysis of ACT-5::GFP abundance in soluble and insoluble fractions. Lysates from transgenic worms expressing ACT-5::GFP in control and *hsf-1* RNAi conditions were subject to ultracentrifugation in the absence of ATP. Shown is a representative blot from 3 replicate experiments.

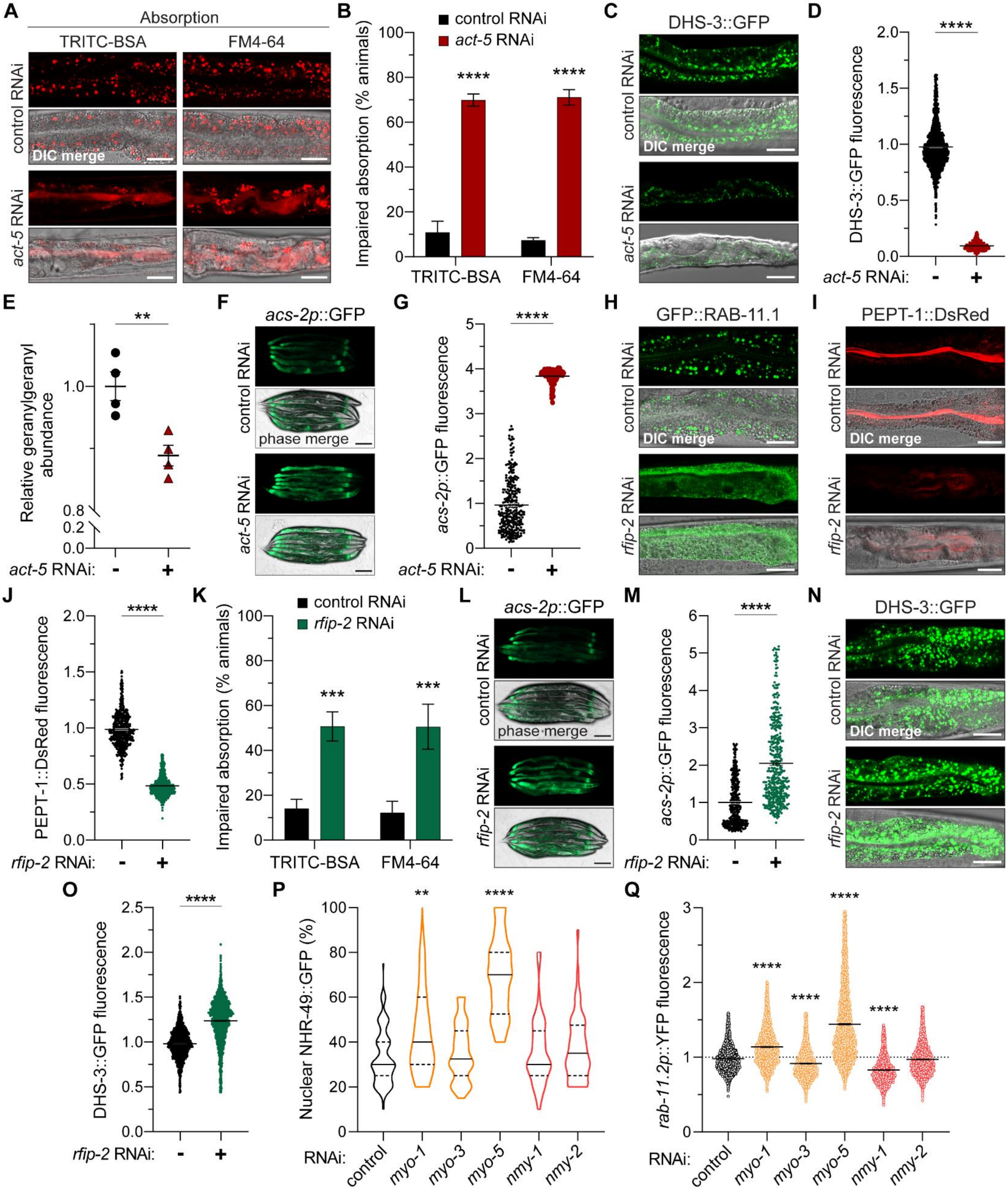

Supplemental Figure S6. Disrupting RAB-11.1 interaction with the enteric actin network activates the intracellular lipid surveillance response. Related to Figure 6. (**A-G**) Comparative analyses of adult *C. elegans* treated with control or *act-5* RNAi. (**A,B**) Absorption of TRITC-BSA, left, or FM4-64, right, into the intestinal epithelia. **A**, Representative confocal micrographs. Scale = 25 µm. **B**, Percentage of animals with impaired

absorption. Mean ± SEM for 3 replicate experiments (100 animals per condition). ****$p < 0.0001$ by two-tailed unpaired t-tests. (**C,D**) DHS-3::GFP expression in day 2 adults. **C**, Representative confocal micrographs. Scale = 25 µm. **D**, Relative fluorescence by flow cytometry. Mean ± SEM for 2187 (control) and 3019 (*act-5*) animals from 3 replicate experiments. ****$p < 0.0001$ by two-tailed unpaired t-test. (**E**) Relative geranylgeranyl levels measured from day 1 adult *C. elegans* cultured at 25°C. Mean ± SEM for 4 replicate experiments. **$p = 0.0078$ by two-tailed unpaired t-test. (**F,G**) Fluorescence of the *acs-2p*::GFP transcriptional reporter. **F**, Fluorescence micrographs. Scale = 200 µm. **G**, Relative fluorescence by flow cytometry. Mean ± SEM for 304 (control) and 363 (*act-5*) animals across 3 replicate experiments. ****$p < 0.0001$ by two-tailed unpaired t-test. (**H-O**) Comparative analyses of adult *C. elegans* treated with control or Rab11 family interacting protein, *rfip-2*, RNAi. (**H**) Representative micrographs of GFP::RAB-11.1 localization. Scale = 25 µm. (**I,J**) PEPT-1::DsRed steady-state expression. **I**, Representative confocal micrographs. Scale = 25 µm. **J**, Relative fluorescence by flow cytometry. Mean ± SEM for 578 (control) and 1,090 (*rfip-2*) animals from 3 replicate experiments. ****$p < 0.0001$ by two-tailed unpaired t-test. (**K**) Percentage of adult *C. elegans* with impaired TRITC-BSA, left, or FM4-64, right, absorption into the intestinal epithelia. Mean ± SEM for 3 replicate experiments (100 animals per condition). ****$p < 0.0001$ by two-tailed unpaired t-tests. (**L,M**) Fluorescence of the *acs-2p*::GFP transcriptional reporter. **L**, Fluorescence micrographs. Scale = 200 µm. **M**, Relative fluorescence by flow cytometry. Mean ± SEM for 419 (control) and 381 (*rfip-2*) animals across 3 replicate experiments. ****$p < 0.0001$ by two-tailed unpaired t-test. (**N,O**) DHS-3::GFP expression in day 3 adults. **N**, Representative confocal micrographs. Scale = 25 µm. **O**, Relative fluorescence by flow cytometry. Mean ± SEM for 1,600 (control) and 2,038 (*rfip-2*) animals from 3 replicate experiments. ****$p < 0.0001$ by two-tailed unpaired t-test. (**P,Q**) Percent of intestinal epithelia with nuclear-enriched NHR-49::GFP, **P**, and relative *rab-11.2p*::YFP reporter fluorescence by flow cytometry, **Q**, in adult *C. elegans* treated with control RNAi or RNAi for various muscle (*myo-1, myo-3, myo-5*) and non-muscle (*nmy-1, nmy-2*) myosins. Median with quartiles for 50 animals per condition across 3 replicate experiments, **P**, and mean ± SEM for, from left to right, 9,119, 5,177, 6,092, 6,159, 3,198 and 6,025 animals across 3 replicate experiments, **Q**. **$p = 0.0013$, ****$p < 0.0001$ by one-way ANOVA with Dunnett's multiple comparisons.

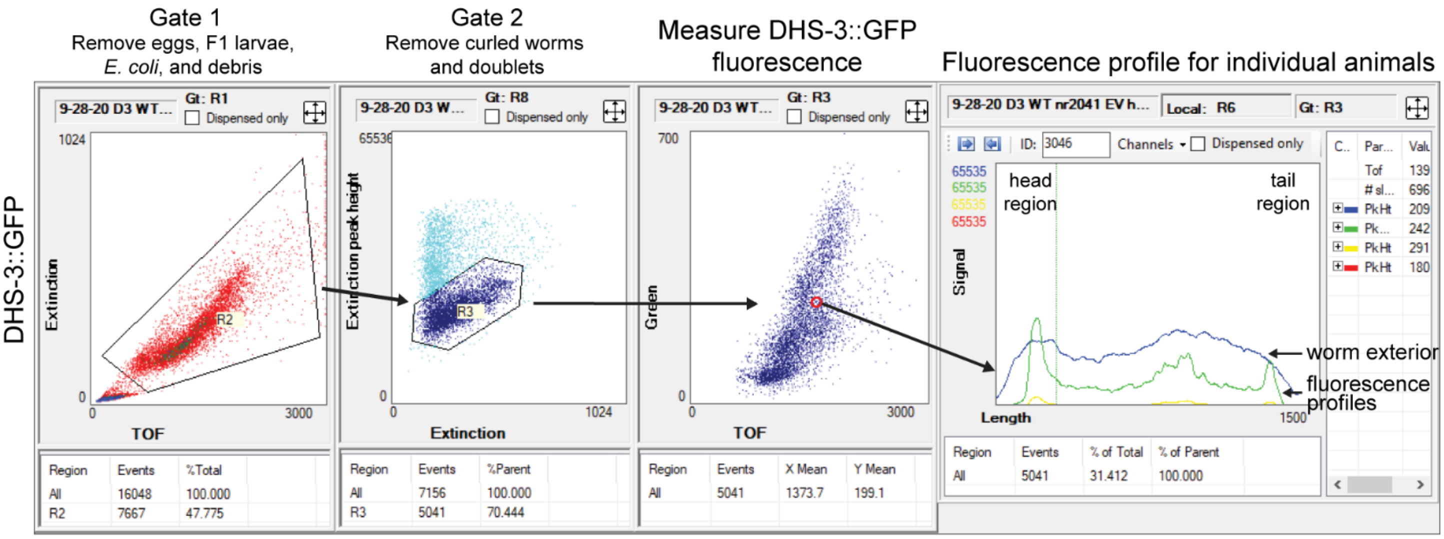

Supplemental Figure S7. Representative gating strategy for large-particle flow cytometry of *C. elegans*. Related to STAR Methods. Shown are representative gating methods to analyze DHS-3::GFP fluorescence in day 3 adult worms, left, and fluorescence profiles for individual worms, right.

Table S1. Fold change and significance of fatty acid and lipid metabolism/mobilization gene expression upon *hsf-1* RNAi and *hsf-1* overexpression (OE) relative to the respective controls. Related to Figures 1E and S1F.

Name	Identifier	*hsf-1* RNAi/control RNAi			*hsf-1* OE/wild-type control		
		Relative fold change	Significance (*p*-value)		Relative fold change	Significance (*p*-value)	
spp-20	K04A8.8	139.00	0.0000	****	-3.93	0.4785	
lipl-8	Y50E8A.7	42.00	0.0003	***	-	-	
fat-5	W06D12.3	12.20	0.0000	****	-1.08	0.7668	
acs-2	F28F8.2	7.58	0.0000	****	-1.22	0.1254	
T28F3.5	T28F3.5	6.95	0.0000	****	-3.76	0.3512	
F42G2.7	F42G2.7	4.00	0.0061	**	-	-	
lea-1	K08H10.1	3.77	0.0000	****	1.13	0.4483	
lipl-3	R11G11.14	3.52	0.0000	****	1.71	0.4940	
Y46H3A.5	Y46H3A.5	3.29	0.0002	***	-2.84	0.1242	
cav-2	C56A3.7	2.92	0.0000	****	1.26	0.0031	**
hacd-1	R09B5.6	2.91	0.0000	****	1.18	0.1503	
cgt-1	T06C12.10	2.81	0.0000	****	1.01	0.8585	
F25A2.1	F25A2.1	2.52	0.0001	***	1.41	0.0304	*
daf-18	T07A9.6	2.46	0.0000	****	-0.62	0.3437	
ZK1251.3	ZK1251.3	-1.68	0.0000	****	-7.99	0.0065	**
abhd-3.2	C44C1.5	-1.77	0.0000	****	-1.14	0.0947	
asah-1	K11D2.2	-1.78	0.0000	****	1.95	0.0007	***
nrf-6	C08B11.4	-1.78	0.0000	****	1.22	0.0023	*
B0478.3	B0478.3	-1.86	0.0044	**	1.49	0.0071	**
F58B6.1	F58B6.1	-2.00	0.0000	****	1.37	0.0450	*
F28H7.3	F28H7.3	-2.04	0.0000	****	1.26	0.0022	**
ech-1.1	C29F3.1	-2.07	0.0000	****	-1.47	0.0994	
W03D8.8	W03D8.8	-2.21	0.0000	****	1.50	0.0117	
ceeh-2	K07C5.5	-2.26	0.0000	****	-1.21	0.0341	*
pgp-2	C34G6.4	-2.57	0.0000	****	1.10	0.4593	
lbp-8	T22G5.6	-3.09	0.0007	***	1.79	0.0067	**
ech-9	F01G10.3	-2.82	0.0000	****	-1.18	0.2851	
gba-4	Y4C6B.6	-3.79	0.0000	****	-1.03	0.1798	
asm-3	W03G1.7	-4.07	0.0000	****	2.12	0.0389	*
acox-3	F58F9.7	-4.10	0.0000	****	-1.30	0.0274	*
vit-2	C42D8.2	-4.25	0.0000	****	7.81	0.1392	
vit-6	K07H8.6	-5.05	0.0000	****	8.68	0.1228	
fat-7	F10D2.9	-5.60	0.0000	****	-2.37	0.0533	
vit-5	C04F6.1	-8.99	0.0000	****	18.94	0.1345	
vit-1	K09F5.2	-9.81	0.0000	****	4.62	0.0759	
vit-3	F59D8.1	-10.87	0.0000	****	-	-	
vit-4	F59D8.2	-12.51	0.0000	****	-	-	

Table S2. Shared differentially regulated genes upon *hsf-1* RNAi and the *nhr-49(nr2041)* mutation. Related to Figure 1F.

**Shared genes (239 total)**					
aagr-1	*clec-218*	*elo-5*	*fat-5*	*nhr-6*	*T24C4.4*
acbp-1	*clec-229*	*elo-9*	*fat-7*	*nlp-26*	*T24E12.5*
acdh-9	*clec-230*	*F08G2.5*	*fbxa-24*	*nlp-29*	*T26H5.9*
acs-2	*clec-242*	*F08G5.6*	*fbxa-91*	*nspc-3*	*T28A11.19*
arf-1.1	*clec-4*	*F09C8.1*	*folt-2*	*nspd-2*	*T28A11.2*
asah-1	*clec-65*	*F15A4.6*	*grl-16*	*nspd-3*	*T28A11.6*
asm-2	*clec-66*	*F15B9.6*	*gst-4*	*oac-10*	*tdc-1*
asm-3	*clec-71*	*F17B5.8*	*gst-5*	*oac-30*	*tsp-1*
asp-10	*clec-72*	*F19C7.4*	*gst-6*	*oac-31*	*tsp-2*
asp-12	*clec-76*	*F20G2.5*	*H20E11.3*	*oac-56*	*ttr-51*
asp-6	*clec-8*	*F22E5.1*	*H43E16.1*	*papl-1*	*tts-1*
B0205.13	*col-120*	*F23A7.4*	*hacd-1*	*pcp-2*	*ugt-10*
B0272.4	*col-152*	*F25E5.8*	*hmit-1.2*	*pgp-1*	*ugt-16*
B0348.2	*col-176*	*F27E5.1*	*hrg-3*	*pmp-5*	*ugt-23*
BE0003N10.6	*col-60*	*F33H12.7*	*ilys-5*	*pqn-91*	*ugt-33*
C08E3.13	*col-71*	*F35F10.1*	*ins-35*	*prx-3*	*ugt-41*
C08E8.4	*col-79*	*F37C12.10*	*irg-1*	*pud-1.2*	*valv-1*
C08F11.13	*cpt-4*	*F41C3.2*	*irg-3*	*pud-2.1*	*vit-1*
C09B8.4	*cpt-5*	*F41G3.10*	*K01D12.8*	*pud-2.2*	*vit-2*
C23G10.6	*ctsa-1*	*F42A10.6*	*K03H6.2*	*pud-3*	*wrt-4*
C23H5.8	*cyp-13A5*	*F43C11.7*	*K07E1.1*	*pud-4*	*Y105C5A.13*
C25F9.11	*cyp-25A1*	*F44G3.10*	*K09C6.9*	*R07B1.13*	*Y105C5B.15*
C32H11.1	*cyp-25A2*	*F45D11.14*	*K09H11.1*	*R08E5.3*	*Y22D7AL.15*
C34B4.2	*cyp-33C8*	*F49C12.7*	*K11H12.11*	*R102.4*	*Y37H2A.14*
C34H4.2	*cyp-35B1*	*F49D11.6*	*klo-2*	*R12E2.14*	*Y41C4A.11*
C36C5.12	*cysl-2*	*F53B2.8*	*lact-4*	*R12E2.15*	*Y47H10A.5*
C36C5.5	*D2045.8*	*F54B11.11*	*lbp-7*	*R12E2.7*	*Y47H9C.1*
C39B5.14	*decr-1.1*	*F54B8.4*	*lips-6*	*scrm-4*	*Y48A6B.7*
C39B5.5	*dgat-2*	*F54D10.8*	*lys-2*	*sodh-1*	*Y51F10.7*
C42D4.1	*dhs-14*	*F54D5.4*	*lys-3*	*spp-3*	*Y51H4A.5*
C42D8.1	*dhs-18*	*F55B11.2*	*M60.4*	*spp-8*	*Y54G2A.10*
C50F7.5	*dhs-25*	*F55C10.4*	*maoc-1*	*srr-4*	*Y73F4A.1*
CC8.2	*dod-17*	*F55G11.6*	*math-14*	*T01D1.4*	*Y75B8A.4*
ceeh-2	*dod-24*	*F55G11.8*	*math-45*	*T10E9.3*	*ZC376.3*
clc-1	*dod-6*	*F58A6.1*	*mboa-3*	*T16G1.6*	*zip-10*
clec-118	*dpm-3*	*F58B4.5*	*mltn-1*	*T19D12.4*	*ZK381.8*
clec-17	*drd-10*	*F58B6.1*	*msra-1*	*T20D4.3*	*ZK512.7*
clec-174	*E02C12.8*	*F58F9.7*	*mtl-2*	*T20D4.4*	*ZK596.1*
clec-186	*ech-1.1*	*F59B1.10*	*mxl-3*	*T20D4.5*	*ZK84.1*
clec-209	*ech-9*	*F59C6.18*	*nhr-155*	*T24B8.5*	

Table S3. Statistics associated with lifespan assays. Related to Figures 2, S2, and 4.

**Figure**	**Treatment group**	**Median lifespan ± SD**	**Observed/ total**	**% change/ control**	***n***	**Log-rank *p*-value**	**Compared to**
S2C	N2, control RNAi	16.7 (±2.1)	297/389	0	3	-	-
S2C	N2, *nhr-49* RNAi	11.7 (±1.2)	304/359	-30	3	< 0.0001	N2, control RNAi
S2C	N2, *hsf-1* RNAi	11.7 (±1.2)	297/356	-30	3	< 0.0001	N2, control RNAi
S2C	N2, *nhr-49* + *hsf-1* RNAi	11 (±0.0)	334/370	-34	3	< 0.0001	N2, *hsf-1* RNAi
2G	N2, control RNAi	16 (±1.0)	315/366	0	3	-	-
2G	N2, *nhr-49* RNAi	11 (±0.0)	294/331	-31.3	3	< 0.0001	N2, control RNAi
2G	AGD710 (*hsf-1* OE), control RNAi	21.3 (±1.5)	304/363	33.3	3	< 0.0001	N2, control RNAi
2G	AGD710 (*hsf-1* OE), *nhr-49* RNAi	11 (±0.0)	327/363	-31.3	3	< 0.0001	AGD710 (*hsf-1* OE), control RNAi
4H	N2, control RNAi	18.5 (±0.7)	261/329	0	2	-	-
4H	N2, *hsf-1* RNAi	12.5 (±0.7)	300/346	-32.4	2	< 0.0001	N2, control RNAi
4H	*rab-11.2(syb2999)*, control RNAi	18.5 (±0.7)	236/287	0	2	0.6760	N2, control RNAi
4H	*rab-11.2(syb2999)*, *hsf-1* RNAi	11.5 (±2.1)	322/349	-37.8	2	< 0.0001	N2, *hsf-1* RNAi

Table S4. ACT-5::GFP interacting proteins with the highest destabilization index values (top 10%). Related to Figure 5D. Represented are the proteins with the largest shift from the soluble fraction in control conditions to the insoluble fraction in *hsf-1* RNAi conditions.

Interacting protein					
ARGK-1	PPM-1	C01G6.3	F54E2.1	ZK1055.7	SEC-23
C18H9.3	RAE-1	C44B7.5	M106.3	HACH-1	SKR-3
T03F6.3	F12F6.7	F59C6.5	Y42H9AR.1	MAOC-1	C33A12.1
DPYD-1	RAL-1	T23G11.7	Y47G6A.18	ZK1320.9	ALH-13
KGB-1	RHO-1	Y38F2AR.12	Y54H5A.2	GCST-1	ABCF-3
C44B7.5	TSR-1	F19B6.1	ZK1307.1	EIF-3.E	FARS-1
ALH-6	TBB-6	EDFY-2	VPS-29	PAR-3	B0395.3
AMPD-1	UFD-3	TPS-1	NAS-20	RUVB-1	CPT-2
F41C3.5	UEV-1	DAF-41	PFKB-1.2	ZK180.4	C29F7.2
K02D7.1	C45G9.5	DNJ-16	ANAT-1	ACDH-11	CDC-37
YARS-1	F22B8.7	MEK-2	T26C12.1	SAMS-1	C29F7.3
F46G10.1	ATIC-1	FNTB-1	C10C5.3	MEN-1	SUCL-2
ACDH-1	ACDH-9	ACS-5	MCA-3	C28G1.5	RPN-8
DPF-4	AMPH-1	FARD-1	C52A10.1	RAB-7	DNJ-13
MVK-1	ARL-8	FCP-1	CDC-42	EDHG-1	PAA-1
RUVB-2	CPZ-1	GST-26	CLEC-85	SDS-22	ACS-13
SEC-24.1	DHRS-4	GST-38	CYN-15	ADS-1	RBP-5
GST-7	ERP-44.1	CDL-1	CHE-10	GLRX-3	F35G12.12
PRMT-1	ECH-5	HMG-12	SAO-1	Y71H2AM.13	PAR-1
C02D5.4	ACS-11	BRE-1	ZK177.8	ZK829.7	TAG-165
C08E8.4	GST-13	RAB-18	SAO-1	IDE-2	MMCM-1
ABCE-1	K02F3.2	GGTB-1	DCS-1	K07C5.2	F53F1.2
ALH-7	SRP-6	Y45G12B.3	UBC-7	Y71G12B.10	UNC-108
CHD-3	TSG-101	MAP-1	SYX-4	SKR-1	TACO-1
EBP-1	Y232F6A.5	MMAA-1	RME-1	EIF-3.K	CPY-35c1
PRX-14	Y47G6A.21	MAPD-1.3	C02C2.6	Y94H6A.10	NUO-1
T08H10.1	Y71H10B.1	HPO-19	UBXN-3	ASB-2	ARP-1
PFK-1.1	PUD-3	ULA-1	UGGT-3	TFG-1	RPN-9
ELB-1	K10C2.1	NPP-1	F21D5.5	CSN-5	GSPD-1
ECH-7	DHS-14	GST-6	C31E10.6	EMB-8	C30G12.2
TBH-1	DPM-1	PAS-3	F31D4.9	B0205.6	EIF-3.L
SDHB-1	ELC-1	PCM-1	W03F8.4	ASNA-1	F26H9.5
Y25C1A.13	Y48A6B.9	F42H10.6	W04B5.5	T20B12.7	UFL-1
APA-2	EIF-1	R151.8A	Y37E3.11	Y56A31.7	C42C1.11
ALH-4	FIPR-21	REP-1	Y43F8C.13	RIL-1	ZC434.8
C06A6.4	UNC-57	RME-1	Y54G11A.7	SDHA-2	AOS-1
NEX-2	R102.4	SPL-1	C27H6.8	STL-1	NUO-4
BICD-1	MTHF-1	UBXN-1	VACL-14	DHS-9	CTL-1
OSTB-1	LIPL-5	C47B2.2	GLDC-1	B0250.5	PPH-5
W09H1.5	CYN-13	D1054.8	TBA-4	GMPS-1	RAB-5
IFE-1	ZK673.2	F10A3.17	NUBP-1	UBA-2	GDI-1
ACS-1	SNX-6	F38A5.2	CTPS-1	IFA-2	CIF-1
UNC-18	OTUB-1	F39B2.3	Y18D10A.11	C05C10.3	
TAX-6	C05D11.1	F41G3.6	GCS-1	AIN-2	

Table S5. Worm strains associated with figures. Related to STAR Methods.

Strain	Description	Figures	Supplemental figures
N2	wild-type	1A, 1B, 1F, 2A, 2B, 2D, 2E, 2G, 2H, 3C, 4C, 4D, 4G, 4H	S1A, S1B, S1C, S1F, S1H, S2C, S3A, S4A, S4B, S4C, S4D, S4G, S4H, S4I
AGD710	*hsf-1* OE (all tissues)	1B, 2D, 2E, 2G, 2H	S1C
LIU1	DHS-3::GFP	1C, 1D, 2C, 2F, 4E, 4F	S2A, S2B, S6C, S6D, S6N, S6O
PMD157	DHS-3::GFP;*hsf-1* OE	1C, 1D, 2F	S2B
PMD121	wild-type	1F	S1H
PMD122	*nhr-49(nr2041)*	1F	S1H
WBM170	*acs-2p*::GFP	1H, 1I	S1K, S1L, S6F, S6G, S6L, S6M
PMD168	*acs-2p*::GFP;*hsf-1* OE	1H, 1I	
STE68	*nhr-49(nr2041)*	2A, 2B	S2C
PMD142	DHS-3::GFP;*nhr-49(nr2041)*	2C	S2A
PMD150	NHR-49::GFP	3A, 3B, 6B, 6F	S6P
PMD124	*rab-11.2p*::YFP	3D, 3E, 6C, 6D, 6G, 6H, 6I	S6Q
PMD118	*rab-11.2p*::YFP;*nhr-49(nr2041)*	3D, 3E, 6C, 6D, 6G, 6H	
RT327	GFP::RAB-5	3F, 6A	
RT476	GFP::RAB-7	3F, 6A	
RT525	GFP::RAB-10	3F, 6A	
RT311	GFP::RAB-11.1	3F, 6A	S3B, S6H
PMD90	GFP::RAB-11.1 (sterile at 25°C)	3G, 5E	S3C
CF512	sterile at 25°C	3H	S6A, S6B, S6E, S6K
MZE1	PEPT-1::DsRed	4A, 4B	S6I, S6J
PMD169	PEPT-1::DsRed;*rab-11.2(syb2999)*	4A, 4B	
PHX2999	*rab-11.2(syb2999)*	4C, 4D, 4G, 4H	S4H, S4I
PMD170	DHS-3::GFP;*rab-11.2(syb2999)*	4E, 4F	
PMD17	ACT-5::mCherry	5A	
PMD19	ACT-5::GFP	5B, 5C	S5A, S5B, S5C
PMD91	GFP::RAB-10 (sterile at 25°C)	5E	
CL2070	*hsp-16.2p*::GFP		S1I,J
OG472	PGP-3::mCherry		S4E, S4F
